# Identification of a New Promising BAG3 Modulator Featuring the Imidazopyridine Scaffold

**DOI:** 10.3390/molecules29215051

**Published:** 2024-10-25

**Authors:** Dafne Ruggiero, Emis Ingenito, Eleonora Boccia, Vincenzo Vestuto, Maria Rosaria Miranda, Stefania Terracciano, Gianluigi Lauro, Giuseppe Bifulco, Ines Bruno

**Affiliations:** 1 Department of Pharmacy, University of Salerno, Via Giovanni Paolo II 132, 84084 Fisciano, Italy; druggiero@unisa.it (D.R.); eingenito@unisa.it (E.I.); eboccia@unisa.it (E.B.); vvestuto@unisa.it (V.V.); mmiranda@unisa.it (M.R.M.); sterracciano@unisa.it (S.T.); glauro@unisa.it (G.L.); 2 PhD Program in Drug Discovery and Development, University of Salerno, Via Giovanni Paolo II 132, 84084 Fisciano, Italy

**Keywords:** BAG3 protein, BAG domain modulator, Groebke–Blackburn–Bienaymé reaction, imidazopyridine scaffold, Surface Plasmon Resonance assay

## Abstract

The antiapoptotic BAG3 protein plays a crucial role in cellular proteostasis and it is involved in several signalling pathways governing cell proliferation and survival. Owing to its multimodular structure, it possesses an extensive interactome including the molecular chaperone HSP70 and other specific cellular partners, which make it an eminent factor in several pathologies, particularly in cancer. Despite its potential as a therapeutic target, very few BAG3 modulators have been disclosed so far. Here we describe the identification of a promising BAG3 modulator able to bind the BAG domain of the protein featuring an imidazopyridine scaffold and obtained through the application of the Groebke–Blackburn–Bienaymé chemical synthesis procedure. The disclosed compound **10** showed a relevant cytotoxic activity, and in line with the biological profile of BAG3 disruption, it induced the activation of caspase 3 and 9.

## 1. Introduction

The increasingly detailed knowledge of the mechanisms underlying malignant transformation has provided better opportunities for designing appropriate therapeutic interventions. For example, it is well known that cancer cells develop alternative mechanisms to circumvent an inhospitable environment and to assure their survival and invasion capacity. Among these, autophagy and apoptosis play a crucial role in response to stressful stimuli, and it is precisely in this regard that the human Bcl-2-associated athanogene (BAG3) protein comes into play. Indeed, as a co-chaperone of the 70 kDa heat shock protein (HSP70), involving its conserved BAG domain, BAG3 governs protein homeostasis activating autophagy, promoting the clearance of misfolded protein, and suppressing apoptosis, thus becoming a focus of research as a potential target for cancer therapies [1,2,3,4]. Moreover, beyond its interaction with HSP70, BAG3, in virtue of its multidomain architecture, can affect several cellular signaling pathways through protein–protein interactions [5,6,7]. Recently, several coimmunoprecipitation experiments were carried out, and coupling with mass spectrometry provided the identification of a wide range of BAG3 interactors, supporting the involvement of the protein in several crucial physiological and pathological cell processes [8,9,10]. A growing mass of evidence highlighted that BAG3 plays a role in the development, prognosis, and resistance to treatment of over 10 different types of cancer, including the aggressive pancreatic ductal adenocarcinomas, and the high expression of the protein in these cancer cells correlates with increased resistance to chemotherapeutics [11,12]. Although the protein offers various sites for intervention with small molecules, targeting the BAG domain is considered the most effective approach to abolish most of its cancer-related characteristics. However, despite this approach holding promises, until now very few modulators binding the BAG domain of the protein have been disclosed, most of which were provided by our research group, either for the absence of a crystal structure of the human BAG3 and/or co-complexed with inhibitors, or for the particular shape of this domain, which appears, in the murine homologue of the protein, as a shallow cavity surrounded by less structured protein segments [13,14]. Continuing our studies on this interesting co-chaperone and aiming at finding new molecular entities able to interact with BAG3, here we decided to investigate new *N*-based heterocycles, prevalent in natural products and well-known for their chemical and biological importance, as molecular probes to explore the target of interest. Specifically, we here focused on the imidazopyridine heterocyclic system, which has gained remarkable importance over the past few years as a key pharmacophore motif for the development of small molecules with potential medicinal applications [15,16,17]. For obtaining these *N*-bridgehead heterobicycles, we took advantage of the versatile one-pot Groebke–Blackburn–Bienaymé reaction using readily available aldehyde, isocyanide and amidine building blocks. Using this versatile procedure, we succeeded in identifying an interesting compound (**10**) able to bind to the BAG domain of BAG3 (BAG3-BD) [18,19,20].

## 2. Results and Discussion

### 2.1. Synthesis

**Figure 1:**
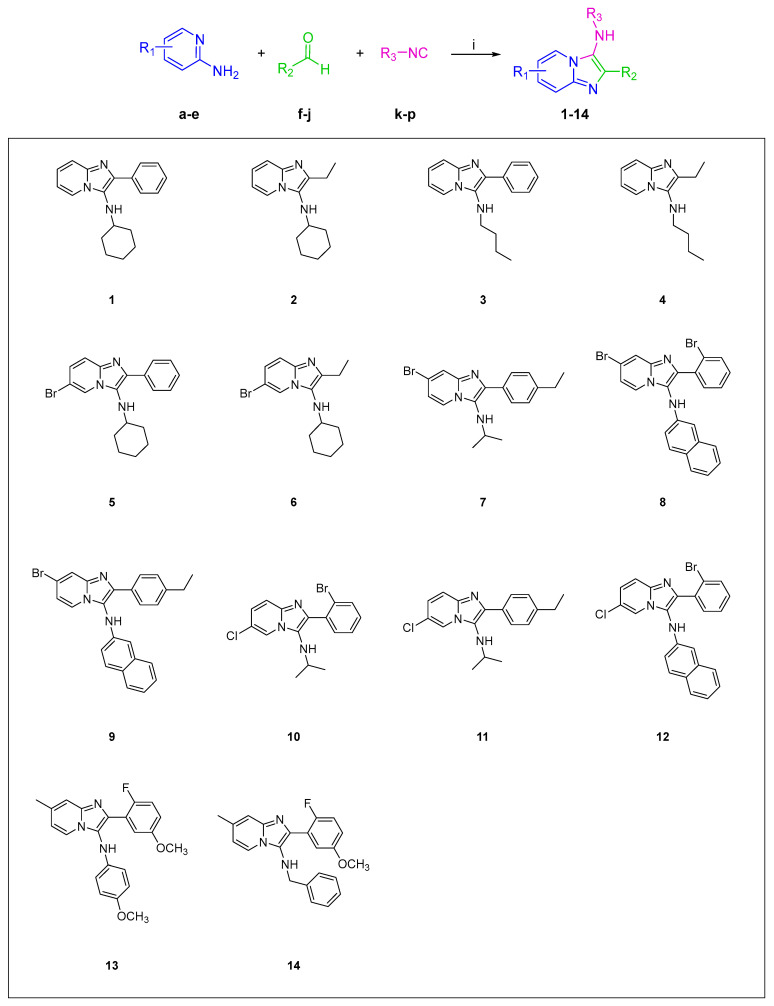

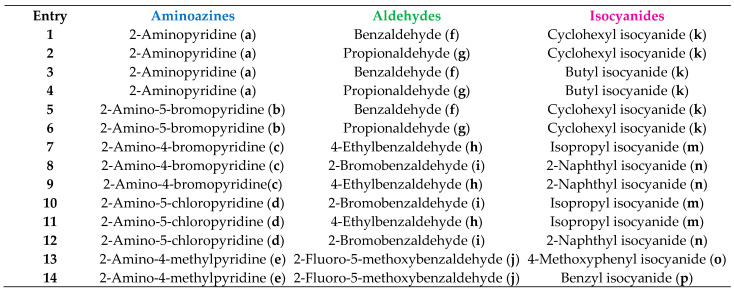
Structures and synthesis of **1**–**14** by Groebke–Blackburn–Bienaymé reaction. Reagents and conditions: (i) MeOH, CH₃COOH, r.t., o.n., HCl, 30 min.

The imidazopyridine scaffold, a fused ring structure combining imidazole and pyridine rings, is a crucial class of heterocyclic compounds in medicinal chemistry. Compounds featuring this scaffold have garnered significant attention due to their broad spectrum of pharmacological activities, including antimicrobial, antiviral, anti-inflammatory, and anticancer properties [21,22,23,24,25,26,27,28]. Their compatibility with drug design principles, such as drug-likeness and synthetic accessibility, further underscores their prominence in the field. A notable method for synthesizing these compounds is the Groebke reaction, a multi-component approach involving the condensation of an aldehyde, an isocyanide, and an aminoazine to form a fused heterocyclic compound [29,30]. The versatility and operational simplicity of the Groebke reaction make this scaffold a valuable tool in drug discovery, facilitating the rapid synthesis of libraries of bioactive compounds. Specifically, we mixed equimolar amounts of aminoazines (**a**–**e**), aldehydes (**f**–**j**), and isocyanides (**k**–**p**) in MeOH and allowed them to react at room temperature overnight. The reaction process starts with imine formation between the aldehyde and aminozole function and involves an electrocyclization with isocyanides. The reaction rate, being pH-dependent, was considerably accelerated by the addition of one to two equivalents of acetic acid [31]. The analysis of the crude reaction mixtures showed complete disappearance of the starting materials in all experiments, enabling us to obtain the desired compounds **1**–**14** with good yields (30–90%) depending on the nature of the isocyanides. The reagents were selected to generate a small collection of compounds displaying a significant structural variability and in order to thoroughly explore the chemical space. Specifically, both aliphatic and aromatic isocyanides were employed, and the same has been undertaken for the selection of aldehydes. The structures of the synthesized compounds are reported in Figure 1.

### 2.2. Biophysical Assays

The Surface Plasmon Resonance (SPR) assay is a sophisticated analytical technique used to study molecular interactions in real time without the need for labels. By measuring changes in the refractive index near a sensor surface, SPR detects binding events between biomolecules, such as proteins, and small molecules [32,33]. 

In this study, we employed SPR experiments to evaluate the interactions between synthesized compounds (**1**–**14**) and both the full-length BAG3 protein and its BAG domain. Only the compounds that successfully bound to the full-length BAG3 protein were tested for their interaction with the BAG domain. Protein immobilization was achieved using the amide coupling method, and the compounds were tested at ten different concentrations, ranging from 0 to 100 µM. A known binder of BAG3-BD, **LK4** ((*Z*)-ethyl 2-(5-(3,4-dihydroxybenzylidene)-2,4-dioxothiazolidin-3yl)acetate), was used as a positive control [13,14]. The assays revealed that three of the fourteen tested compounds have affinity for the target. In particular, compounds **10** and **12** bound both the full-length protein and the domain with K_D_ values comparable to the known inhibitor **LK4** (Table 1). Compound **14** also bound the target but with slightly higher K_D_ values, still within the micromolar range. The three identified compounds were further investigated to understand whether this binding induced an effect on BAG3 protein functions. The K_D_ values reported in Table 1 for the known inhibitor **LK4** differ from those in previous studies due to the use of a newer-generation instrument for the SPR experiments. The earlier experiments utilized the Biacore 3000, whereas the current study employed the Biacore T200. While ligand binding was confirmed in both sets of experiments, the slightly different K_D_ values are attributed to the enhanced sensitivity of the Biacore T200.

### 2.3. Viability Assays

**Figure 2:**
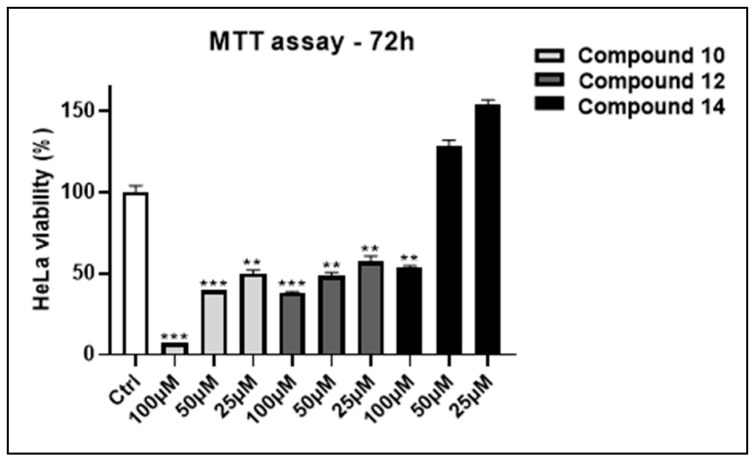
Antiproliferative activity of compounds **10**, **12** and **14** on human cervical adenocarcinoma cell line (HeLa) after 72 h of treatment at 25, 50 and 100 µM. The positive control (CTRL POS) consisted of 10% DMSO, while the negative control (CTRL NEG) consisted of 0.1% DMSO. The results are showed as mean ± standard deviation (SD) from three independent experiments. **, *** denote respectively *p* < 0.01 and *p* < 0.001 vs. Ctrl.

Elevated expression levels of BAG3 are strongly associated with signaling pathways in various types of malignant cells; consequently, reducing the activity of this co-chaperone inhibits cell proliferation, triggers apoptosis, and induces cell cycle arrest [34]. To assess the activity of compounds **10**, **12**, and **14**, cytotoxicity assays were performed on HeLa cells, which were chosen for their tendency to overexpress BAG3 proteins [14,35]. MTT assays were conducted by exposing HeLa cells to different concentrations of the compounds (25, 50, and 100 µM) for 72 h. Additionally, 10% DMSO was used as a positive control, while 0.1% DMSO served as a negative control [33]. All three compounds exhibited cytotoxic effects at 100 µM: compound **10** induced over 90% mortality, compound **12** caused 60% mortality, and compound **14** resulted in 50% mortality. As the concentration decreased, the effects changed; at 25 µM, only compounds **10** and **12** remained cytotoxic, with 50% and 40% mortality rates, respectively, while compound **14** lost its effect. Hence, we determined the IC_50_ values for compounds **10** and **12** by testing them at seven different concentrations (ranging from 1 to 100 µM) on HeLa cells over 72 h (IC_50_-**10**—25.11 ± 2.67 µM; IC_50_-**12**—38.85 ± 3.49 µM) (Figure 2). The next step involved the assessment of the cytotoxicity of the most promising candidate, **10**, on healthy cells to determine its safety profile. To achieve this, MTT assays were conducted on HaCat cells over 72 h. The cells were exposed to seven different concentrations of the compound (ranging from 1 to 100 μM) to establish the IC_50_, which was determined to be 81.30 ± 4.83 μM. These findings suggest that compound **10** can be considered an initial seed for the next optimization process, as it is more toxic to tumor cells while affecting healthy cells only at higher concentrations.

### 2.4. Biological Assays

**Figure 3:**
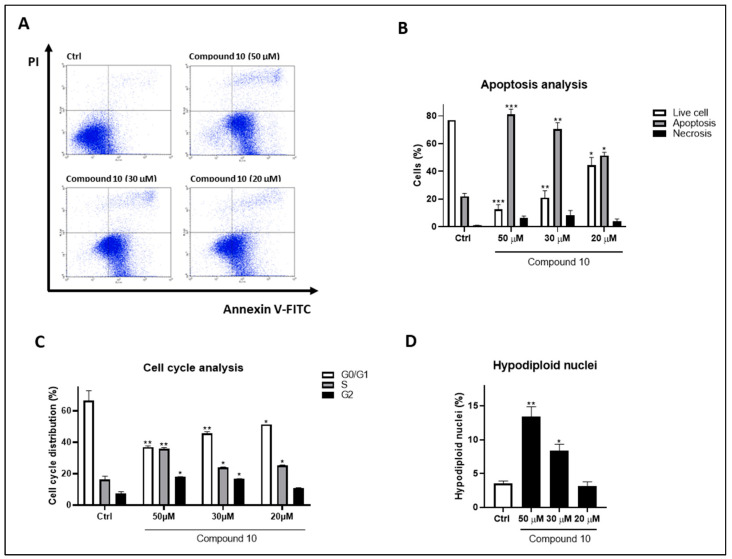
Pro-apoptotic activity on HeLa cells treated for 72 h with compound **10** at 50, 30 and 20 μM. (**A**) Representative flow cytometry plots using Annexin V-FITC/PI staining for apoptosis. (**B**) Related quantitative analysis is reported. Data are expressed as a percentage of live, apoptotic and necrotic cells. (**C**) Cell cycle analysis. (**D**) Hypodiploid nuclei determination. Results are shown as mean ± standard deviation (SD) from three independent experiments. *, **, *** denote, respectively, *p* < 0.05; *p* < 0.01 and *p* < 0.001 vs. Ctrl.

Given BAG3’s well-documented anti-apoptotic role, annexin V-FITC/PI staining was performed to evaluate the effects of compound **10** on cell apoptosis. Fluorescence-activated cell sorting (FACS) analysis indicated that the treatment of HeLa cells with compound **10** induced apoptosis in a concentration-dependent manner (50–20 µM) [36,37]. Specifically, at 50 µM, 81.0 ± 3.89% of the cells were apoptotic (*p* < 0.001 vs. Ctrl); at 30 µM, 70.5 ± 4.56% were apoptotic (*p* < 0.01 vs. Ctrl); and at 20 µM, 51.5 ± 2.23% were apoptotic (*p* < 0.05 vs. Ctrl) (Figure 3A,B). Additionally, consistent and significant cell cycle arrest as well as an increase in hypodiploid nuclei was observed across all tested concentrations (Figure 3C,D).

**Figure 4:**
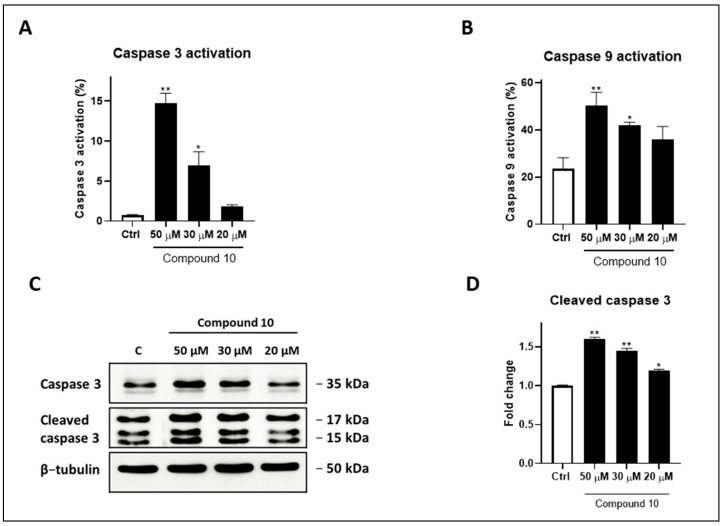
Activation of caspases on HeLa cells treated for 72 h with compound **10** at 50, 30 and 20 μM. (**A**) Caspase 3 and (**B**) caspase 9 determination via cytofluorimetric technique. (**C**) Western blot analyses. Levels of cleaved caspase were evaluated. Βactin was used to check the equal loading of protein extracts. (**D**) Densitometric analysis of western blots. Results are shown as mean ± standard deviation (SD) from three independent experiments. *, ** denote, respectively, *p* < 0.05 and *p* < 0.01 vs. Ctrl.

Furthermore, we investigated the expressions of caspases, key mediators of programmed cell death, in HeLa cells treated with compound **10** at the same concentrations used in the previous assays. Flow cytometry revealed a pronounced, dose-dependent activation of both caspase 3 and caspase 9 in cells exposed to compound **10** compared to the control (Figure 4A,B) [14]. To further confirm the pro-apoptotic mechanism induced by compound **10**, Western blotting was performed to measure the expression and cleavage of caspase 3 [38,39]. The results demonstrate an increased expression of procaspase 3, along with elevated levels of cleaved caspase 3 in a dose-dependent manner (50 µM: *p* < 0.01 vs. Ctrl; 30 µM: *p* < 0.01 vs. Ctrl; 20 µM: *p* < 0.05 vs. Ctrl), indicating the activation of apoptosis and aligning with the annexin V-FITC/PI data (Figure 4C,D).

### 2.5. Computational Studies

**Figure 5:**
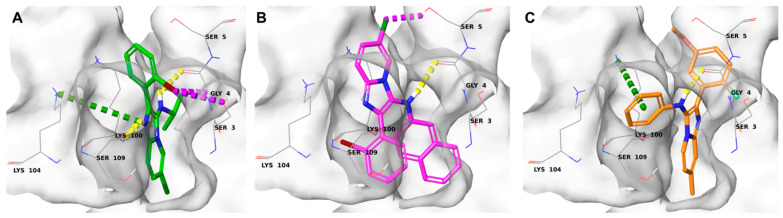
Binding mode of (**A**) compound **10** (colored by atom type: C green, N blue, Cl dark green, Br dark red, polar H white), (**B**) compound **12** (colored by atom type: C pink, N blue, Cl dark green, Br dark red, polar H white), (**C**) compound **14** (colored by atom type: C orange, N blue, O red, Br dark red, Cl dark green, polar H white). H bonds, halogen bonds, and π interactions are reported in yellow, purple and green dotted lines, respectively.

To corroborate the data obtained for the most promising compounds (**10**, **12**, and **14**) and to rationalize their binding modes at the molecular level with the protein of interest, molecular docking studies were conducted against BAG3. Specifically, the crystal structure of the murine BAG-binding domain of BAG3 (PDB entry: 1UK5) was used, due to the lack of a human co-crystallized structure with substrates and/or inhibitors. Given the absence of precise information on the fundamental amino acids of the binding site, docking poses of the active compounds **10**, **12**, and **14** were visually inspected and compared to detect the ability of the tested compounds to establish relevant interaction patterns within the binding site. As shown in Figure 4, the docking results disclosed that all three compounds occupy the same binding site region, specifically interacting through hydrogen bonds with the Gly4 residue. A detailed analysis of the docking poses also highlighted additional interactions for each compound: compound **10** (Figure 5A) binds the counterpart via its imidazopyridine moiety through π-interactions with the Lys104 residue, additional halogen interactions with the Gly4 and Ser3, and a hydrogen bond with Ser109; compound **12** (Figure 5B) also interacts with Ser5 through halogen bonds; compound **14** forms π-interactions with Lys100. 

## 3. Materials and Methods

### 3.1. Synthetic Procedure 

All starting materials and solvents used in this study were obtained from Merck (Darmstadt, Germany). NMR spectra (^1^H and ^13^C) were recorded on Bruker Avance instruments operating at field strengths of 400, 500, and 600 MHz, at a temperature of 298 K (Bruker, Milan, Italy). The compounds were dissolved in 0.5 mL of CD_3_OD or CDCl_3_ (Merck, 99.8 Atom %D). Coupling constants (*J*) are reported in Hertz, and chemical shifts are given in parts per million (ppm) on the delta (δ) scale, relative to the solvent peak as the internal reference. Multiplicities are denoted as follows: s (singlet), d (doublet), dd (doublet of doublets), ddd (doublet of doublet of doublets), td (triplet of doublets), t (triplet), dt (doublet of triplets), q (quartet), and m (multiplet). Mass spectrometry experiments were conducted using an LTQ Orbitrap XL mass spectrometer (Thermo Scientific, Monza, Italy). Reaction monitoring was performed on silica gel 60 F254 plates (Merck), with spot visualization under UV light at wavelengths of 254 nm and 365 nm. Semi-preparative reversed-phase HPLC was conducted using an Agilent Technologies 1200 Series high-performance liquid chromatography system with a Synergi Fusion C18 reversed-phase column (250 × 10.0 mm, 4 μm, 80 Å) at a flow rate of 4 mL/min (Phenomenex). The binary solvent system (A/B) consisted of 0.1% TFA in water (A) and 0.1% TFA in CH3CN (B), with absorbance detection at 240 nm. All biologically tested compounds were confirmed to be >98% pure by HPLC analysis and NMR data.

*General procedure for the synthesis of compounds* **1**–**14**

Amine **a**–**e** (1 mmol), aldehyde **f**–**j** (1 mmol), and isocyanide **k**–**p** (1 mmol) were dissolved or suspended in 3 mL of MeOH. Glacial acetic acid (2 mmol) was then added to the mixture. The reaction was stirred at room temperature overnight. The mixture was subsequently acidified with 1N HCl to a pH of 1 and stirred for an additional 30 min to destroy any residual isocyanide. The solvent was then evaporated to dryness. The resulting residue was taken up in aqueous KHCO_3_ and extracted with EtOAc. Further purification was achieved through column chromatography and through HPLC chromatography [31,40]. See Supplementary Materials for all spectral data (Figures S1–S42).

*N-cyclohexyl-2-phenylimidazo[1,2-a]pyridin-3-amine* (**1**)

Compound **1** was obtained by following the general procedure from the reaction between 2-aminopyridine (**a**), benzaldehyde (**f**) and cyclohexyl isocyanide (**k**) as a white powder (175.7 mg, 97% yield after HPLC purification).

RP-HPLC t_R_ = 22 min, gradient condition: from 5% B ending to 100% B 40 min, flow rate of 4 mL/min, λ = 240 nm.

^1^H NMR (600 MHz, Methanol-*d*_4_): δ = 8.76 (d, *J* = 6.8 Hz, 1H), 8.00–7.95 (m, 3H), 7.87 (d, *J* = 8.9 Hz, 1H), 7.63 (t, *J* = 7.6 Hz, 2H), 7.59–7.51 (m, 2H), 3.01–2.95 (m, 1H), 1.86–1.80 (m, 2H), 1.73–1.66 (m, 2H), 1.60–1.54 (m, 1H), 1.33–1.25 (m, 2H), 1.23–1.13 (m, 3H).

^13^C NMR (151 MHz, Methanol-*d*_4_): δ = 137.0, 133.0, 129.7, 129.0 (2C), 127.3 (2C), 126.9 (2C), 126.5, 125.0, 116.6, 111.4, 56.2, 33.4 (2C), 25.3, 24.4 (2C).

ESI-MS: calculated for C_19_H_21_N_3_ 291.1735; found *m*/*z* = 292.1815 [M + H]^+^.

*N-cyclohexyl-2-ethylimidazo[1,2-a]pyridin-3-amine* (**2**)

Compound **2** was obtained by following the general procedure from the reaction between 2-aminopyridine (**a**), propionaldehyde (**g**) and cyclohexyl isocyanide (**k**) as a white powder (167 mg, 82% yield after HPLC purification).

RP-HPLC t_R_ = 19 min, gradient condition: from 5% B ending to 100% B 40 min, flow rate of 4 mL/min, λ = 240 nm.

^1^H NMR (400 MHz, Methanol-*d*_4_): δ = 8.51 (dt, *J* = 6.8, 1.1 Hz, 1H), 7.80 (ddd, *J* = 9.0, 7.0, 1.2 Hz, 1H), 7.70 (dt, *J* = 9.0, 1.1 Hz, 1H), 7.37 (td, *J* = 6.9, 1.2 Hz, 1H), 2.92–2.78 (m, 3H), 1.91–1.79 (m, 2H), 1.78–1.67 (m, 2H), 1.60–1.49 (m, 1H), 1.34–1.10 (m, 8H).

^13^C NMR (101 MHz, Methanol-*d*_4_): δ = 136.7, 132.2, 130.0, 126.6, 124.8, 116.2, 111.2, 56.1, 33.6 (2C), 25.4, 24.6 (2C), 16.9, 11.9.

ESI-MS: calculated for C_15_H_21_N_3_ 243.1735; found *m*/*z* = 244.1814 [M + H]^+^.

*N-butyl-2-phenylimidazo[1,2-a]pyridin-3-amine* (**3**)

Compound **3** was obtained by following the general procedure from the reaction between 2-aminopyridine (**a**), benzaldehyde (**f**) and butyl isocyanide (**k**). Purification by silica gel (8:2 hexane/ethyl acetate) and then an HPLC purification gave a compound in the form of a white powder (195 mg, 60% yield after HPLC purification).

RP-HPLC t_R_ = 21 min, gradient condition: from 5% B ending to 100% B 40 min, flow rate of 4 mL/min, λ = 240 nm.

^1^H NMR (600 MHz, Methanol-*d*_4_): δ = 8.73 (d, *J* = 6.8 Hz, 1H), 7.99–7.90 (m, 3H), 7.87 (d, *J* = 8.9 Hz, 1H), 7.63 (t, *J* = 7.5 Hz, 2H), 7.55 (dt, *J* = 19.3, 7.1 Hz, 2H), 3.08 (t, *J* = 7.2 Hz, 2H), 1.59–1.52 (m, 2H), 1.43–1.35 (m, 2H), 0.89 (t, *J* = 7.4 Hz, 3H).

^13^C NMR (151 MHz, Methanol-*d*_4_): δ = 136.8, 132.9, 129.7, 129.0 (2C), 128.3, 127.3 (2C), 126.8, 125.2, 124.8, 116.6, 111.4, 46.8, 32.2, 19.7, 12.7.

ESI-MS: calculated for C_17_H_19_N_3_ 265.1579; found *m*/*z* = 266.1657 [M + H]^+^.

*N-butyl-2-ethylimidazo[1,2-a]pyridin-3-amine* (**4**)

Compound **4** was obtained by following the general procedure from the reaction between 2-aminopyridine (**a**), propionaldehyde (**g**) and butyl isocyanide (**k**). Purification by silica gel (7:3 ethyl acetate/hexane) and then HPLC purification gave a compound in the form of a yellow oil (119 mg, 45% yield after HPLC purification).

RP-HPLC t_R_ = 16 min, gradient condition: from 5% B ending to 100% B 40 min, flow rate of 4 mL/min, λ = 240 nm.

^1^H NMR (600 MHz, Methanol-*d*_4_): δ = 8.49–8.47 (m, 1H), 7.77 (ddd, *J* = 8.4, 7.0, 1.2 Hz, 1H), 7.67 (dt, *J* = 9.0, 1.1 Hz, 1H), 7.35 (td, *J* = 6.9, 1.1 Hz, 1H), 2.97 (t, *J* = 7.2 Hz, 2H), 2.82 (q, *J* = 7.6 Hz, 2H), 1.54–1.46 (m, 2H), 1.38 (dt, *J* = 15.0, 7.4 Hz, 2H), 1.28 (t, *J* = 7.6 Hz, 3H), 0.87 (t, *J* = 7.4 Hz, 3H).

^13^C NMR (151 MHz, Methanol-*d*_4_): δ = 136.5, 132.2, 129.0, 128.0, 124.6, 116.3, 111.2, 32.4, 19.7, 16.8 (2C), 12.8, 12.1.

ESI-MS: calculated for C_13_H_19_N_3_ 217.1579; found *m*/*z* = 218.1658 [M + H]^+^.

*6-bromo-N-cyclohexyl-2-phenylimidazo[1,2-a]pyridin-3-amine* (**5**)

Compound **5** was obtained by following the general procedure from the reaction between 2-amino-5-bromopyridine (**b**), benzaldehyde (**f**) and cyclohexyl isocyanide (**k**) as a yellowish powder (117 mg, 99% yield after HPLC purification).

RP-HPLC t_R_ = 24 min, gradient condition: from 5% B ending to 100% B 40 min, flow rate of 4 mL/min, λ = 240 nm.

^1^H NMR (600 MHz, Methanol-*d*_4_): δ = 8.89 (d, *J* = 2.2 Hz, 1H), 8.00 (dd, *J* = 9.4, 1.8 Hz, 1H), 7.97–7.94 (m, 2H), 7.77 (d, *J* = 9.4 Hz, 1H), 7.62 (dd, *J* = 8.3, 6.7 Hz, 2H), 7.59–7.55 (m, 1H), 2.99–2.94 (m, 1H), 1.85–1.78 (m, 2H), 1.73–1.66 (m, 2H), 1.60–1.54 (m, 1H), 1.33–1.23 (m, 2H), 1.21–1.12 (m, 3H).

^13^C NMR (151 MHz, Methanol-*d*_4_): δ = 135.9, 135.1, 135.0, 129.8, 128.9 (2C), 127.3 (2C), 127.2, 127.1, 124.9, 112.7, 110.8, 56.2, 33.4 (2C), 25.2, 24.4 (2C).

ESI-MS: calculated for C_19_H_20_BrN_3_ 369.0841; found *m*/*z* = 370.0923 [M + H]^+^.

*6-bromo-N-cyclohexyl-2-ethylimidazo[1,2-a]pyridin-3-amine* (**6**)

Compound **6** was obtained by following the general procedure from the reaction between 2-amino-5-bromopyridine (**b**), propionaldehyde (**g**) and cyclohexyl isocyanide (**k**) as a white powder (139 mg, 63% yield after HPLC purification).

RP-HPLC t_R_ = 22 min, gradient condition: from 5% B ending to 100% B 40 min, flow rate of 4 mL/min, λ = 240 nm.

^1^H NMR (400 MHz, Methanol-*d*_4_): δ = 8.65 (dd, *J* = 1.8, 0.9 Hz, 1H), 7.89 (dd, *J* = 9.5, 1.8 Hz, 1H), 7.65 (dd, *J* = 9.4, 0.9 Hz, 1H), 2.94–2.77 (m, 3H), 1.90–1.79 (m, 2H), 1.75–1.65 (m, 2H), 1.60–1.51 (m, 1H), 1.34–1.08 (m, 8H).

^13^C NMR (101 MHz, Methanol-*d*_4_): δ = 135.3, 135.1, 131.0, 126.9, 124.8, 112.3, 110.9, 56.4, 33.5 (2C), 25.3, 24.6 (2C), 17.0, 11.7.

ESI-MS: calculated for C_15_H_20_BrN_3_ 321.0841; found *m*/*z* = 322.0919 [M + H]^+^.

*7-bromo-2-(4-ethylphenyl)-N-isopropylimidazo[1,2-a]pyridin-3-amine* (**7**)

Compound **7** was obtained by following the general procedure from the reaction between 2-amino-4-bromopyridine (**c**), 4-ethylbenzaldehyde (**h**) and isopropyl isocyanide (**m**) as a yellowish powder (98 mg, 98% yield after HPLC purification).

RP-HPLC t_R_ = 32 min, gradient condition: from 5% B ending to 100% B 40 min, flow rate of 4 mL/min, λ = 240 nm.

^1^H NMR (500 MHz, Methanol-*d*_4_): δ = 8.28 (d, *J* = 7.2 Hz, 1H), 7.97 (d, *J* = 7.8 Hz, 2H), 7.68 (s, 1H), 7.33 (d, *J* = 7.8 Hz, 2H), 7.09–7.04 (m, 1H), 3.33–3.28 (d, *J* = 6.3 Hz, 1H), 2.72 (q, *J* = 7.6 Hz, 2H), 1.33–1.26 (m, 3H), 1.10–1.03 (m, 6H).

^13^C NMR (126 MHz, Methanol-*d*_4_): δ = 143.8, 141.2 (2C), 136.6, 130.9, 127.6 (2C), 127.0 (2C), 123.8, 118.0, 117.7, 115.3, 48.4, 28.2, 22.0 (2C), 14.7.

ESI-MS: calculated for C_18_H_20_BrN_3_ 357.0841; found *m*/*z* = 358.0916 [M + H]^+^.

*7-bromo-2-(2-bromophenyl)-N-(naphthalen-2-yl)imidazo[1,2-a]pyridin-3-amine* (**8**)

Compound **8** was obtained by following the general procedure from the reaction between 2-amino-4-bromopyridine (**c**), 2-bromobenzaldehyde (**i**) and 2-naphthyl isocyanide (**n**). Purification by silica gel (1:1 ethyl acetate/hexane) and then HPLC purification gave a compound in the form of a pink powder (145 mg, 30% yield after HPLC purification).

RP-HPLC t_R_ = 24 min, gradient condition: from 5% B ending to 100% B 40 min, flow rate of 4 mL/min, λ = 240 nm.

^1^H NMR (500 MHz, Methanol-*d*_4_): δ = 7.99 (d, *J* = 7.2 Hz, 1H), 7.90–7.86 (m, 1H), 7.67 (dt, *J* = 8.2, 1.8 Hz, 3H), 7.52 (dd, *J* = 7.7, 1.8 Hz, 1H), 7.47 (d, *J* = 8.3 Hz, 1H), 7.36 (td, *J* = 7.5, 1.2 Hz, 1H), 7.31 (ddd, *J* = 8.2, 6.8, 1.3 Hz, 1H), 7.26 (td, *J* = 7.7, 1.7 Hz, 1H), 7.21 (ddd, *J* = 8.1, 6.8, 1.2 Hz, 1H), 7.13 (dd, *J* = 7.2, 1.9 Hz, 1H), 7.01 (dd, *J* = 8.8, 2.4 Hz, 1H), 6.64 (d, *J* = 2.4 Hz, 1H).

^13^C NMR (126 MHz, Methanol-*d*_4_): δ = 142.6, 141.9, 138.9, 134.8, 134.0, 132.7, 131.8, 129.8, 128.9, 128.5, 127.1, 126.9, 125.9, 125.7, 124.0, 123.3, 122.4, 121.5, 119.3, 118.6, 116.6, 116.2, 106.5.

ESI-MS: calculated for C_23_H_15_Br_2_N_3_ 490.9633; found *m*/*z* = 491.9703 [M + H]^+^.

*7-bromo-2-(4-ethylphenyl)-N-(naphthalen-2-yl)imidazo[1,2-a]pyridin-3-amine* (**9**)

Compound **9** was obtained by following the general procedure from the reaction between 2-amino-4-bromopyridine (**c**), 4-ethylbenzaldehyde (**h**) and 2-Naphthyl isocyanide (**n**). Purification by silica gel (7:3 hexane/ethyl acetate) and then HPLC purification gave a compound in the form of a yellowish powder (385 mg, 35% yield after HPLC purification).

RP-HPLC t_R_ = 33 min, gradient condition: from 5% B ending to 100% B 40 min, flow rate of 4 mL/min, λ = 240 nm.

^1^H NMR (600 MHz, Methanol-*d*_4_): δ = 7.84–7.81 (m, 1H), 7.81–7.78 (m, 2H), 7.74–7.71 (m, 1H), 7.69 (d, *J* = 8.0 Hz, 1H), 7.64 (d, *J* = 8.8 Hz, 1H), 7.59 (d, *J* = 8.2 Hz, 1H), 7.34 (d, *J* = 8.5 Hz, 1H), 7.19 (ddd, *J* = 8.2, 6.8, 1.3 Hz, 1H), 7.13–7.08 (m, 3H), 6.98–6.92 (m, 2H), 6.51 (d, *J* = 2.4 Hz, 1H), 2.52 (q, *J* = 7.6 Hz, 2H), 1.09 (t, *J* = 7.6 Hz, 3H).

^13^C NMR (151 MHz, Methanol-*d*_4_): δ = 144.4, 142.9, 142.5, 139.1, 135.0, 129.9, 129.4 (2C), 128.6, 127.7 (2C), 127.2, 126.8 (2C), 126.1, 125.7, 123.7, 122.4, 119.3, 118.3, 116.3, 116.0, 106.1, 28.2, 14.6.

ESI-MS: calculated for C_25_H_20_BrN_3_ 441.0841; found *m*/*z* = 442.0912 [M + H]^+^.

*2-(2-bromophenyl)-6-chloro-N-isopropylimidazo[1,2-a]pyridin-3-amine* (**10**)

Compound **10** was obtained by following the general procedure from the reaction between 2-amino-5-chloropyridine (**d**), 2-bromobenzaldehyde (**i**) and isopropyl isocyanide (**m**) as a brownish powder (228 mg, 47% yield after HPLC purification).

RP-HPLC t_R_ = 28 min, gradient condition: from 5% B ending to 100% B 40 min, flow rate of 4 mL/min, λ = 240 nm.

^1^H NMR (600 MHz, Methanol-*d*_4_): δ = 8.80 (d, *J* = 2.2 Hz, 1H), 7.91–7.84 (m, 2H), 7.84–7.79 (m, 1H), 7.69 (dd, *J* = 7.6, 1.8 Hz, 1H), 7.60 (td, *J* = 7.5, 1.2 Hz, 1H), 7.53 (td, *J* = 7.8, 1.8 Hz, 1H), 3.24–3.17 (m, 1H), 1.04 (d, *J* = 6.3 Hz, 6H).

^13^C NMR (151 MHz, Methanol-*d*_4_): δ = 135.3, 133.1, 132.6, 132.5, 131.9, 129 (2C), 127.8, 124.6, 124.3, 124.0, 122.6, 113.0, 47.6, 21.9 (2C).

ESI-MS: calculated for C_16_H_15_BrClN_3_ 363.0138; found *m*/*z* = 364.0213 [M + H]^+^.

*6-chloro-2-(4-ethylphenyl)-N-isopropylimidazo[1,2-a]pyridin-3-amine* (**11**)

Compound **11** was obtained by following the general procedure from the reaction between 2-amino-5-chloropyridine (**d**), 4-ethylbenzaldehyde (**h**) and isopropyl isocyanide (**m**) as a white powder (117 mg, 37% yield after HPLC purification).

RP-HPLC t_R_ = 33 min, gradient condition: from 5% B ending to 100% B 40 min, flow rate of 4 mL/min, λ = 240 nm.

^1^H NMR (600 MHz, Methanol-*d*_4_): δ = 8.40 (dd, *J* = 2.1, 0.9 Hz, 1H), 7.97–7.93 (m, 2H), 7.47 (dd, *J* = 9.5, 0.9 Hz, 1H), 7.31 (d, *J* = 8.1 Hz, 2H), 7.26 (dd, *J* = 9.4, 2.0 Hz, 1H), 3.32–3.29 (m, 1H), 2.71 (q, *J* = 7.6 Hz, 2H), 1.29 (t, *J* = 7.6 Hz, 3H), 1.06 (d, *J* = 6.3 Hz, 6H).

^13^C NMR (151 MHz, Methanol-*d*_4_): δ = 143.9, 139.6, 137.1, 131.0, 127.6 (2C), 127.0 (2C), 126.2, 125.4, 120.8, 120.1, 116.4, 48.4, 28.1, 22.0 (2C), 14.5.

ESI-MS: calculated for C_18_H_20_ClN_3_ 313.1346; found *m*/*z* = 314.1419 [M + H]^+^.

*2-(2-bromophenyl)-6-chloro-N-(naphthalen-2-yl)imidazo[1,2-a]pyridin-3-amine* (**12**)

Compound **12** was obtained by following the general procedure from the reaction between 2-amino-5-chloropyridine (**d**), 2-bromobenzaldehyde (**i**) and 2-naphthyl isocyanide (**n**). Purification by silica gel (7:3 hexane/ethyl acetate) and then HPLC purification gave the compound as a brownish powder (188 mg, 30% yield after HPLC purification).

RP-HPLC t_R_ = 32 min, gradient condition: from 5% B ending to 100% B 40 min, flow rate of 4 mL/min, λ = 240 nm.

^1^H NMR (500 MHz, Chloroform-*d*): δ = 7.97–7.93 (m, 1H), 7.71 (dt, *J* = 10.3, 5.3 Hz, 3H), 7.64 (dd, *J* = 8.1, 1.2 Hz, 1H), 7.59 (dd, *J* = 7.7, 1.7 Hz, 1H), 7.53 (d, *J* = 8.3 Hz, 1H), 7.38 (ddd, *J* = 8.2, 6.8, 1.3 Hz, 1H), 7.33 (td, *J* = 7.5, 1.3 Hz, 1H), 7.30–7.26 (m, 2H), 7.22 (td, *J* = 7.7, 1.8 Hz, 1H), 6.91 (dd, *J* = 8.8, 2.4 Hz, 1H), 6.62 (d, *J* = 2.4 Hz, 1H), 5.92 (s, 1H).

^13^C NMR (126 MHz, Chloroform-*d*): δ = 141.7, 140.7, 134.5, 134.2, 133.1, 132.8, 132.3, 130.0, 129.7, 128.7, 127.7, 127.5, 126.7, 126.5, 126.3, 123.3, 123.0, 121.0 (2C), 120.2, 118.5, 116.6, 107.4.

ESI-MS: calculated for C_23_H_15_BrClN_3_ 447.0138; found *m*/*z* = 448.0210 [M + H]^+^.

*2-(2-fluoro-5-methoxyphenyl)-N-(4-methoxyphenyl)-7-methylimidazo[1,2-a]pyridin-3-amine* (**13**)

Compound **13** was obtained by following the general procedure from the reaction between 2-amino-4-methylpyridine (**e**), 2-fluoro-5-methoxybenzaldehyde (**j**) and 4-methoxyphenyl isocyanide (**o**). Purification by silica gel (7:3 hexane/ethyl acetate) and then HPLC purification gave a compound in the form of brownish powder (188 mg, 52% yield after HPLC purification).

RP-HPLC t_R_ = 27 min, gradient condition: from 5% B ending to 100% B 40 min, flow rate of 4 mL/min, λ = 240 nm.

^1^H NMR (600 MHz, Chloroform-*d*): δ = 7.64 (d, *J* = 6.9 Hz, 1H), 7.27 (dd, *J* = 5.8, 3.2 Hz, 1H), 7.19 (s, 1H), 6.94 (t, *J* = 9.6 Hz, 1H), 6.74 (dt, *J* = 9.1, 3.6 Hz, 1H), 6.67–6.63 (m, 2H), 6.55 (dd, *J* = 7.0, 1.6 Hz, 1H), 6.38–6.33 (m, 2H), 5.58 (d, *J* = 5.2 Hz, 1H), 3.71 (s, 3H), 3.64 (s, 3H), 2.35 (s, 3H).

^13^C NMR (151 MHz, Chloroform-*d*): δ = 155.9, 155.0, 153.4, 143.1, 138.5, 135.7, 133.2, 122.4, 122.0, 121.0, 116.6, 116.5, 116.1, 116.0, 115.9, 114.9 (2C), 114.8, 114.4, 55.8, 55.7, 21.2.

ESI-MS: calculated for C_22_H_20_FN_3_O_2_ 377.1540; found *m*/*z* = 378.1607 [M + H]^+^.

*N-benzyl-2-(2-fluoro-5-methoxyphenyl)-7-methylimidazo[1,2-a]pyridin-3-amine* (**14**)

Compound **14** was obtained by following the general procedure from the reaction between 2-amino-4-methylpyridine (**e**), 2-fluoro-5-methoxybenzaldehyde (**j**) and benzyl isocyanide (**p**). Purification by silica gel (7:3 hexane/ethyl acetate) and then HPLC purification gave a compound in the form of a yellow powder (150 mg, 98% yield after HPLC purification).

RP-HPLC t_R_ = 31 min, gradient condition: from 5% B ending to 100% B 40 min, flow rate of 4 mL/min, λ = 240 nm.

^1^H NMR (500 MHz, Chloroform-*d*): δ = 8.03 (d, *J* = 7.0 Hz, 1H), 7.33 (s, 1H), 7.31–7.27 (m, 1H), 7.26–7.18 (m, 5H), 7.04 (dd, *J* = 10.3, 8.9 Hz, 1H), 6.85 (dt, *J* = 8.8, 3.6 Hz, 1H), 6.65 (dd, *J* = 7.0, 1.6 Hz, 1H), 4.04 (d, *J* = 5.5 Hz, 2H), 3.87 (s, 3H), 2.42 (s, 3H).

^13^C NMR (126 MHz, Chloroform-*d*): δ = 156.0, 154.8, 153.0, 142.4, 139.1, 134.9, 130.8, 128.4 (2C), 128.1 (2C), 127.4, 122.6, 121.9, 116.2, 115.8, 115.6, 114.5 (2C), 55.9, 52.4, 21.4.

ESI-MS: calculated for C_22_H_20_FN_3_O_2_ 361.1590; found *m*/*z* = 362.1661 [M + H]^+^.

### 3.2. Surface Plasmon Resonance

Recombinant human BAG3 protein (Bcl2-associated athanogene 3) was sourced from Novus Biologicals (Littleton, CO, USA), while the BAG3 domain (BAG3-BD) was procured from ARETA International S.r.l. (Gerenzano, Italy). We utilized (*Z*)-ethyl 2-(5-(3,4-dihydroxybenzylidene)-2,4-dioxothiazolidin-3-yl)acetate (LK4), a known BAG3 inhibitor previously identified by our group [13]. Surface Plasmon Resonance (SPR) spectroscopy was employed to assess the affinity of synthesized molecules for BAG3 and BAG-BD using a Biacore T200 optical biosensor equipped with CM5 sensor chips (Cytiva, Marlborough, MA, USA). BAG3 and BAG-BD were immobilized on the CM5 sensor chip using standard amine-coupling protocols. BAG3 protein (0.067 μg/μL in 10 mM CH_3_COONa, pH 4.5) was immobilized at a flow rate of 10 μL/min, resulting in a density of 14 kRU. The BAG3 domain (0.38 μg/μL in 10 mM CH_3_COONa, pH 4.5) was immobilized under similar conditions, achieving a density of 4 kRU. For the experiments, BAG3 and BAG-BD surfaces, along with an unmodified reference surface, were prepared for simultaneous analyses.

Compounds **1**–**14** and **LK4** were dissolved to 10 mM in 100% DMSO and then diluted 1:20 (*v*/*v*) in PBS-P buffer (0.2 M phosphate buffer, 27 mM KCl, 1.37 M NaCl, 0.5% surfactant P20) to achieve a final DMSO concentration of 5.0%. These compounds were injected in a concentration series (1:2 dilution, 10 different concentrations) ranging from 0 to 100 μM, prepared in 96-well plates. SPR experiments were conducted at 25 °C with a flow rate of 20 μL/min, featuring 90 s of association and 400 s of dissociation monitoring. Changes in mass, reflected as resonance units (RU), were recorded to determine binding responses. K_D_ values were calculated using Biaevaluation software 3.2 by globally fitting the double-referenced association and dissociation data to a 1:1 interaction model [14,33]. See Supplementary Materials for all the sensorgrams (Figures S43–S50).

### 3.3. Cell Culture

Human cervical carcinoma cell lines (HeLa, kindly donated by Prof. Ornella Moltedo, Department of Pharmacy, University of Salerno) and immortalized human keratinocytes (HaCaT) were cultured in a DMEM/high glucose medium. This medium was supplemented with 10% FBS (*v*/*v*), 2 mM/L glutamine, 100 U/mL penicillin, and 100 mg/mL streptomycin. The cells were maintained at 37 °C in a humidified atmosphere with 5% CO_2_. To ensure logarithmic growth, the cells were sub-cultured every 2 days.

### 3.4. Cell Viability

Cell viability was assessed by measuring mitochondrial metabolic activity using a colorimetric assay, which involves the reduction of 3-[4,5-dimethylthiazol-2,5-diphenyl-2H-tetrazolium bromide (MTT) to purple formazan. Stock solutions of compounds 10, 12, and 14 (50 mM in DMSO) were stored at −20 °C in the dark and diluted just before being added to the sterile culture medium. Briefly, HeLa cells (3.5 × 10^3^ cells/well) were seeded in triplicate in 96-well plates, and after 24 h, to allow for attachment, the cells were treated with various concentrations of the tested compounds (25, 50 and 100 μM), 0.1% (*v*/*v*) DMSO as a negative control, and 10% (*v*/*v*) DMSO as a positive control for 72 h. Following treatment, 20 μL of MTT (5 mg/mL in PBS) was added to each well, and the cells were incubated for an additional 3 h at 37 °C. The formazan crystals formed were then dissolved in 100 μL of a buffer containing 50% (*v*/*v*) N, N-dimethylformamide and 20% SDS (pH 4.5). Absorbance was measured at 570 nm using a Multiskan™ GO Microplate Spectrophotometer (Thermo Fisher Scientific, Waltham, MA, USA,). For compounds **10** and **12**, which showed significant antiproliferative activity on HeLa cells, proliferation assays were also conducted at seven different concentrations (ranging from 1.56 μM to 100 μM) to determine the IC_50_ value after 72 h. The IC_50_ values, representing the concentration causing 50% inhibition of cell survival, were calculated and compared to control cells treated with DMSO using GraphPad Prism 8.0 software through nonlinear regression of dose–response inhibition. Only for compound 10 was the IC_50_ also assessed on HaCaT cells (5.0 × 10^3^ cells/well) after 72 h of treatment [14,33].

### 3.5. Apoptosis and Cell Cycle Analysis

Hypodiploid nuclei were analyzed using PI staining by flow cytometry. HeLa cells (1 × 10^5^ cells/well) were grown in 12-well plates and allowed to adhere for 24 h. Subsequently, the medium was replaced with fresh medium, and the cells were treated for 72 h with compound **10** at concentrations of 20, 30, and 50 µM, 0.1% (*v*/*v*) DMSO (negative control), and 10% (*v*/*v*) DMSO (positive control). Following treatment, the culture medium was replaced, and the cells were washed twice with PBS. The cells were then suspended in 500 mL of a solution containing 50 mg/L PI, 0.1% (*w*/*v*) sodium citrate, and 0.1% Triton X-100. Both the culture medium and PBS were centrifuged, and the resulting cell pellets were combined with the cell suspension to ensure the retention of both dead and living cells for analysis. After incubation at 4 °C for 30 min in the dark, cell nuclei were analyzed using a Becton Dickinson FACS flow cytometer (Franklin Lakes, NJ, USA) with the Cell Quest program, and the DNA content of the nuclei was recorded. Cellular debris was excluded from the analysis by increasing the forward scatter threshold, and the percentages of cells in the hypodiploid region (sub-G0/G1), G1, S, and G2 regions were calculated [14]. See Supplementary Materials for the FACS histograms (Figure S51). 

### 3.6. Caspase 3 and Caspase 9 Expression

HeLa cells were seeded into 6-well plates (2.5 × 10^5^ cells/well) and allowed to adhere for 24 h. The cells were then treated for 72 h with compound **10** at concentrations of 20, 30, and 50 µM, as well as with 0.1% DMSO negative (control), to assess caspase 3 and caspase 9 levels. After treatment, cells were collected, washed with PBS, and incubated at 4 °C first with a fixing buffer (containing 1% formaldehyde and 1% FBS in PBS) for 20 min, followed by permeabilization with fix perm solution (fixing buffer containing 0.1% Triton X-100) for 30 min. Next, the cells were incubated with anti-caspase 3 (1:500; SC-7272, Santa Cruz, CA, USA) and anti-caspase 9 (1:50; BS-0049R, Bioss Antibodies, Woburn, MA, USA) antibodies for 30 min at 4 °C. Subsequently, cells were incubated with anti-mouse (1:50; A90-116D3, Bethyl, Montgomery, AL, USA) or anti-rabbit (A120-101D2, Bethyl, Montgomery, AL, USA) secondary antibodies for 30 min at 4 °C. Cell fluorescence was then evaluated using a fluorescence-activated cell sorter (FACSscan, Becton Dickinson, Milan, Italy) and analyzed with Cell Quest software (4.0, Becton Dickinson, North Ryde, Australia). Data were presented as a percentage of cells treated with 0.1% DMSO alone [14]. See Supplementary Materials for the FACS histograms (Figures S52 and S53). 

### 3.7. Western Blot

The HeLa cell line was seeded in 60 mm culture dishes (20 × 104) and treated with compound 10 (50, 30, 20 μM) for 72 h. After treatments, the cells were washed twice, detached with a scraper, and centrifuged for 10 min (400× *g*, 4 °C) to remove debris. Total proteins were extracted by lysis buffer (20 mM Tris-HCl pH 7.5, 150 mM NaCl, 1 mM Na2EDTA, 1 mM EGTA, 2% NP-40, 1% sodium deoxycholate, 1× protease, and phosphatase inhibitor cocktail) for 30 min. Thereafter, cell lysates were centrifuged at 18,800× *g* for 15 min at 4 °C. Protein concentration was determined by the Bradford assay using bovine serum albumin as standard. Then, 40 μg of total proteins was run on 12% SDS-PAGE and transferred to nitrocellulose membranes using a minigel apparatus (Bio-Rad Laboratories, Richmond, BC, Canada). Ponceau S dye was applied, and the proteins on the membrane were stained with a red color, after which the membrane was destained with MQ water. Blots were blocked in phosphate buffered saline, containing Tween-20 0.1% and 10% nonfat dry milk for 1 h at room temperature and incubated overnight with specific primary antibodies at 4 °C with slight agitation. Rabbit polyclonal anti-caspase 3 (1:1000, Cell signaling) was used. Mouse monoclonal anti-β-Tubulin (1:1000, Sigma Aldrich, Darmstadt, Germany) was used as the loading control.

After washes in PBS/Tween-20 0.1% the appropriate anti-rabbit or anti-mouse (1:5000, Pierce, Thermo Fisher Scientific) peroxidase-linked secondary antibody was added for 1 h at room temperature. Antigen–antibody complexes were detected through enhanced chemiluminescence using LAS 4000 (GE Healthcare, Chicago, IL, USA) and a densitometry analysis of the autoradiographs was performed by using the ImageJ program, version 1.47 [38,39]. See Supplementary Materials for the Western blots raw data (Figure S55). 

### 3.8. Annexin V-FITC/PI Staining

Apoptosis of the cells was assessed using the Annexin V-FITC/PI reagents. Hela cells (20 × 10^3^) were seeded into 24-well plates and incubated for 72 h with compound 10. After treatment, the collected cells were resuspended in a 100 μL assay buffer, then 5 μL Annexin V-FITC and 1 μL PI reagents were added following an incubation for 20 min at RT according to the manufacturer’s protocol (Dead Cell Apoptosis Kits with Annexin V for Flow Cytometry, Thermo Fisher Scientific). The cells were analyzed with a Becton Dickinson FACScan flow cytometer using the Cell Quest software, version 4 (Franklin Lakes, NJ, USA). See Supplementary Materials for the data plots (Figure S54). 

### 3.9. Statistical Analysis

The data are here reported as mean ± SD of the results from three independent experiments. Statistical analysis was performed using an analysis of variance test (ANOVA), and multiple comparisons were made with the Bonferroni test using GraphPad Prism 8.0 software (San Diego, CA, USA). Significance was assumed at *p* < 0.05.

### 3.10. Computational Details

The 3D structure of the murine BAG domain of Bcl2-associated athanogene 3 (PDB: 1UK5) was downloaded from the Protein Data Bank and was prepared using the Schrödinger Protein Preparation Wizard workflow (Schrödinger Suite) [41]. Specifically, water molecules were removed, cap termini were included, all hydrogen atoms were added, and bond orders were assigned. The grid accounted for the subsequent molecular docking calculations generated considering the whole surface of the protein (due to the absence of known BAG3 modulators and the small size of the protein). The final coordinates of the grid center were 0.0 Å (x); −8.0 Å (y); and 3.0 Å (z) with inner and outer box dimensions of 40 Å and 46 Å, respectively.

The 2D structures of compounds **10**, **12**, and **14** were drawn employing the Maestro 2D sketcher tool and then prepared using LigPrep software (version 5.7, Schrödinger, LLC, New York, NY, USA, 2021) [42,43]: all the possible tautomers and protonation states at pH = 7.4 ± 1.0 were generated, and then the resulting structures were minimized using an OPLS 2005 force field. Molecular docking calculations were performed through Glide software (version 9.0, Schrödinger, LLC, New York, NY, USA, 2021) at the Standard Precision (SP) level, setting 10,000 poses for the initial phase for energy minimization, 5.0 kcal/mol as the energy window for ring sampling, 400.0 as the scoring window for keeping the initial poses, 0.8 as the scaling factor related to van der Waals radii, and 0.15 as a partial charge cutoff based on a 0.5 kcal/mol rejection cutoff for the obtained minimized poses. Finally, a maximum of 50 maximum poses was considered for the subsequent analysis and visual inspections [44].

## 4. Conclusions

Despite the progressive unravelling of BAG3’s biological role and its increasing attractiveness as a therapeutic target in cancer, very few modulators have been identified so far. This is largely due to several challenges, most notably the absence of a crystallographic structure for hBAG3. Consequently, alternative approaches have been pursued to achieve the crucial goal of identifying modulators for this protein. In this study we succeeded in identifying an interesting BAG3 modulator able to bind the BAG domain of the protein, featuring an imidazopyridine scaffold obtained through the application of the Groebke–Blackburn–Bienaymé procedure. The disclosed molecule showed promising cytotoxic activity on HeLa, and, in line with the inhibition of the BAG3 protein, induced apoptosis as well as the activation of caspases 3 and 9. This discovery is particularly important because it expands the range of molecular structures capable of interacting with the protein, and adds new information to the very limited arsenal of molecules already known to modulate BAG3, which represents an attractive candidate for anticancer drug development.

## Figures and Tables

**Table 1:** SPR assays of compounds **1**–**14** and **LK4** on BAG3 full-length and BAG3-BD. K_D_ = dissociation constant; SD = standard deviation. The structure of **LK4** is shown below.

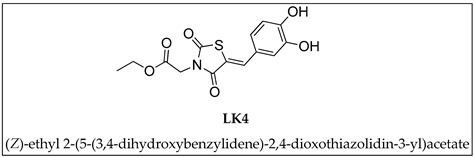		
Compound	BAG3 FULL K_D_ (µM) ± SD	BAG DOMAIN K_D_ (µM) ± SD
**1**	n.b. ^a^	n.d. ^b^
**2**	n.b.	n.d.
**3**	n.b.	n.d.
**4**	n.b.	n.d.
**5**	n.b.	n.d.
**6**	n.b.	n.d.
**7**	n.b.	n.d.
**8**	n.b.	n.d.
**9**	n.b.	n.d.
**10**	33.5 ± 0.21	15.7 ± 1.44
**11**	n.b.	n.d.
**12**	20.70 ± 0.42	14.70 ± 2.69
**13**	n.b.	n.d.
**14**	76.20 ± 5.44	16.90 ± 5.87
**LK4**	19.90 ± 6.58	0.18 ± 0.02

^a^ n.b. = no binding; ^b^ n.d. = not determined.

## Data Availability

Data are contained within the article and Supplementary Materials.

## Supplementary Materials

The following supporting information can be downloaded at: https://www.mdpi.com/article/10.3390/molecules29215051/s1, Figure S1: Compound 1 ^1^H NMR (600 MHz, Methanol-d4); Figure S2: Compound 1 ^13^C NMR (151 MHz, Methanol-d4); Figure S3: Compound 1 ESI-MS spectrum; Figure S4: Compound 2 ^1^H NMR (400 MHz, Methanol-d4); Figure S5: Compound 2 ^13^C NMR (101 MHz, Methanol-d4); Figure S6: Compound 2 ESI-MS spectrum: Figure S7: Compound 3 ^1^H NMR (600 MHz, Methanol-d4); Figure S8: Compound 3 ^13^C NMR (151 MHz, Methanol-d4); Figure S9: Compound 3 ESI-MS spectrum; Figure S10: Compound 4 ^1^H NMR (600 MHz, Methanol-d4); Figure S11: Compound 4 ^13^C NMR (151 MHz, Methanol-d4); Figure S12: Compound 4 ESI-MS spectrum; Figure S13: Compound 5 ^1^H NMR (600 MHz, Methanol-d4); Figure S14: Compound 5 ^13^C NMR (151 MHz, Methanol-d4); Figure S15: Compound 5 ESI-MS spectrum; Figure S16: Compound 6 ^1^H NMR (400 MHz, Methanol-d4); Figure S17: Compound 6 ^13^C NMR (101 MHz, Methanol-d4); Figure S18: Compound 6 ESI-MS spectrum; Figure S19: Compound 7 ^1^H NMR (500 MHz, Methanol-d4); Figure S20: Compound 7 ^13^C NMR (126 MHz, Methanol-d4); Figure S21: Compound 7 ESI-MS spectrum; Figure S22. Compound 8 ^1^H NMR (500 MHz, Methanol-d4); Figure S23: Compound 8 ^13^C NMR (126 MHz, Methanol-d4); Figure S24. Compound 8 ESI-MS spectrum; Figure S25: Compound 9 ^1^H NMR (600 MHz, Methanol-d4); Figure S26: Compound 9 ^13^C NMR (151 MHz, Methanol-d4); Figure S27: Compound 9 ESI-MS spectrum; Figure S28: Compound 10 ^1^H NMR (600 MHz, Methanol-d4); Figure S29: Compound 10 ^13^C NMR (151 MHz, Methanol-d4); Figure S30: Compound 10 ESI-MS spectrum; Figure S31: Compound 11 ^1^H NMR (600 MHz, Methanol-d4); Figure S32: Compound 11 ^13^C NMR (151 MHz, Methanol-d4); Figure S33: Compound 11 ESI-MS spectrum; Figure S34: Compound 12 ^1^H NMR (500 MHz, Chloroform-d); Figure S35: Compound 12 ^13^C NMR (126 MHz, Chloroform-d); Figure S36: Compound 12 ESI-MS spectrum; Figure S37: Compound 13 ^1^H NMR (600 MHz, Chloroform-d); Figure S38: Compound 13 ^13^C NMR (151 MHz, Chloroform-d); Figure S39: Compound 13 ESI-MS spectrum; Figure S40: Compound 14 ^1^H NMR (500 MHz, Chloroform-d) S30; Figure S41: Compound 14 ^13^C NMR (126 MHz, Chloroform-d); Figure S42: Compound 14 ESI-MS spectrum; Figure S43: Sensorgram of compound 10 on BAG3 full length protein; Figure S44: Sensorgram of compound 12 on BAG3 full length protein; Figure S45: Sensorgram of compound 14 on BAG3 full length protein; Figure S46: Sensorgram of LK4 on BAG3 full length protein; Figure S47: Sensorgram of compound 10 on BAG3-BD; Figure S48. Sensorgram of compound 12 on BAG3-BD; Figure S49: Sensorgram of compound 14 on BAG3-BD; Figure S50: Sensorgram of LK4 on BAG3-BD; Figure S51: FACS plots and histograms for cell cycle analysis; Figure S52: FACS histograms for caspase 3 analysis; Figure S53: FACS histograms for caspase 9 analysis; Figure S54: Data plot of apoptotic analysis of compound 10 on HeLa cells using AnnexinV-FITC/PI staining; Figure S55: Western blots raw data of caspase 3 and β-tubulin after treatments with compound 10 on HeLa cells.
