# Supplementary material for: Identification of a New Promising BAG3 Modulator Featuring the Imidazopyridine Scaffold
Source: Molecules. 2024 Oct 25;29(21):5051. doi: 10.3390/molecules29215051 (PMC11547576; doi:10.3390/molecules29215051)
Supplement: Supplementary file 1 [file molecules-29-05051-s001.zip › molecules-3274064-supplementary.pdf]

Supporting Information

Identification of a New Promising BAG3 Modulator Featuring the Imidazopyridine Scaffold

Dafne Ruggiero [1], Emis Ingenito [1,2], Eleonora Boccia [1,2], Vincenzo Vestuto [1], Maria Rosaria Miranda [1,2], Stefania Terracciano [1], Gianluigi Lauro [1], Giuseppe Bifulco [1,*] and Ines Bruno [1,*]

[1] Department of Pharmacy, University of Salerno, Via Giovanni Paolo II 132, 84084 Fisciano, Italy; druggiero@unisa.it (D.R.); eingenito@unisa.it (E.I.); eboccia@unisa.it (E.B.); vvestuto@unisa.it (V.V.); mmiranda@unisa.it (M.R.M.); sterracciano@unisa.it (S.T.); glauro@unisa.it (G.L.)

[2] PhD Program in Drug Discovery and Development, University of Salerno, Via Giovanni Paolo II 132, 84084 Fisciano, Italy

* Correspondence: bifulco@unisa.it (G.B.); brunoin@unisa.it (I.B.); Tel.: +39-089-969-741 (G.B.); +39-089-969-743 (I.B.)

Summary

Figure S1. Compound 1 ^{1}H NMR (600 MHz, Methanol-d4) S4
Figure S2. Compound 1 ^{13}C NMR (151 MHz, Methanol-d4) S4
Figure S3. Compound 1 ESI-MS spectrum S5
Figure S4. Compound 2 ^{1}H NMR (400 MHz, Methanol-d4) S6
Figure S5. Compound 2 ^{13}C NMR (101 MHz, Methanol-d4) S6
Figure S6. Compound 2 ESI-MS spectrum S7
Figure S7. Compound 3 ^{1}H NMR (600 MHz, Methanol-d4) S8
Figure S8. Compound 3 ^{13}C NMR (151 MHz, Methanol-d4) S8
Figure S9. Compound 3 ESI-MS spectrum S9
Figure S10. Compound 4 ^{1}H NMR (600 MHz, Methanol-d4) S10
Figure S11. Compound 4 ^{13}C NMR (151 MHz, Methanol-d4) S10
Figure S12. Compound 4 ESI-MS spectrum S11
Figure S13. Compound 5 ^{1}H NMR (600 MHz, Methanol-d4) S12
Figure S14. Compound 5 ^{13}C NMR (151 MHz, Methanol-d4) S12
Figure S15. Compound 5 ESI-MS spectrum S13
Figure S16. Compound 6 ^{1}H NMR (400 MHz, Methanol-d4) S14
Figure S17. Compound 6 ^{13}C NMR (101 MHz, Methanol-d4) S14
Figure S18. Compound 6 ESI-MS spectrum S15
Figure S19. Compound 7 ^{1}H NMR (500 MHz, Methanol-d4) S16
Figure S20. Compound 7 ^{13}C NMR (126 MHz, Methanol-d4) S16
Figure S21. Compound 7 ESI-MS spectrum S17
Figure S22. Compound 8 ^{1}H NMR (500 MHz, Methanol-d4) S18
Figure S23. Compound 8 ^{13}C NMR (126 MHz, Methanol-d4) S18
Figure S24. Compound 8 ESI-MS spectrum S19
Figure S25. Compound 9 ^{1}H NMR (600 MHz, Methanol-d4) S20
Figure S26. Compound 9 ^{13}C NMR (151 MHz, Methanol-d4) S20
Figure S27. Compound 9 ESI-MS spectrum S21
Figure S28. Compound 10 ^{1}H NMR (600 MHz, Methanol-d4) S22
Figure S29. Compound 10 ^{13}C NMR (151 MHz, Methanol-d4) S22
Figure S30. Compound 10 ESI-MS spectrum S23
Figure S31. Compound 11 ^{1}H NMR (600 MHz, Methanol-d4) S24
Figure S32. Compound 11 ^{13}C NMR (151 MHz, Methanol-d4) S24
Figure S33. Compound 11 ESI-MS spectrum S25
Figure S34. Compound 12 ^{1}H NMR (500 MHz, Chloroform-d) S26
Figure S35. Compound 12 ^{13}C NMR (126 MHz, Chloroform-d) S26
Figure S36. Compound 12 ESI-MS spectrum S27

Figure S37. Compound 13 ^{1}H NMR (600 MHz, Chloroform-d)........ S28
Figure S38. Compound 13 ^{13}C NMR (151 MHz, Chloroform-d)........ S28
Figure S39. Compound 13 ESI-MS spectrum........ S29
Figure S40. Compound 14 ^{1}H NMR (500 MHz, Chloroform-d)........ S30
Figure S41. Compound 14 ^{13}C NMR (126 MHz, Chloroform-d)........ S30
Figure S42. Compound 14 ESI-MS spectrum........ S31
Figure S43. Sensorgram of compound 10 on BAG3 full length protein........ S32
Figure S44. Sensorgram of compound 12 on BAG3 full length protein........ S32
Figure S45. Sensorgram of compound 14 on BAG3 full length protein........ S32
Figure S46. Sensorgram of LK4 on BAG3 full length protein........ S33
Figure S47. Sensorgram of compound 10 on BAG3-BD........ S34
Figure S48. Sensorgram of compound 12 on BAG3-BD........ S34
Figure S49. Sensorgram of compound 14 on BAG3-BD........ S34
Figure S50. Sensorgram of LK4 on BAG3-BD........ S35
Figure S51. FACS plots and histograms for cell cycle analysis........ S36
Figure S52. FACS histograms for caspase 3 analysis........ S37
Figure S53. FACS histograms for caspase 9 analysis........ S37
Figure S54. Data plot of apoptotic analysis of compound 10 on HeLa cells using AnnexinV-FITC/PI staining........ S38
Figure S55. Western blots raw data of caspase 3 and β-tubulin after treatments with compound 10 on HeLa cells........ S39

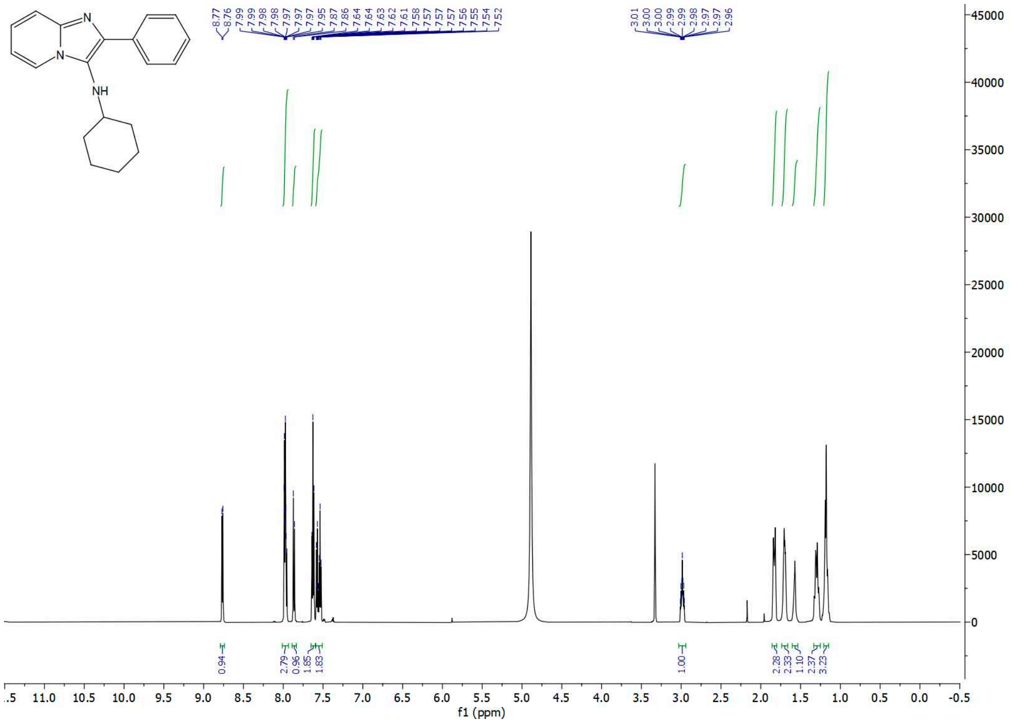

Figure S1. Compound 1 ^{1}H NMR (600 MHz, Methanol-d4)

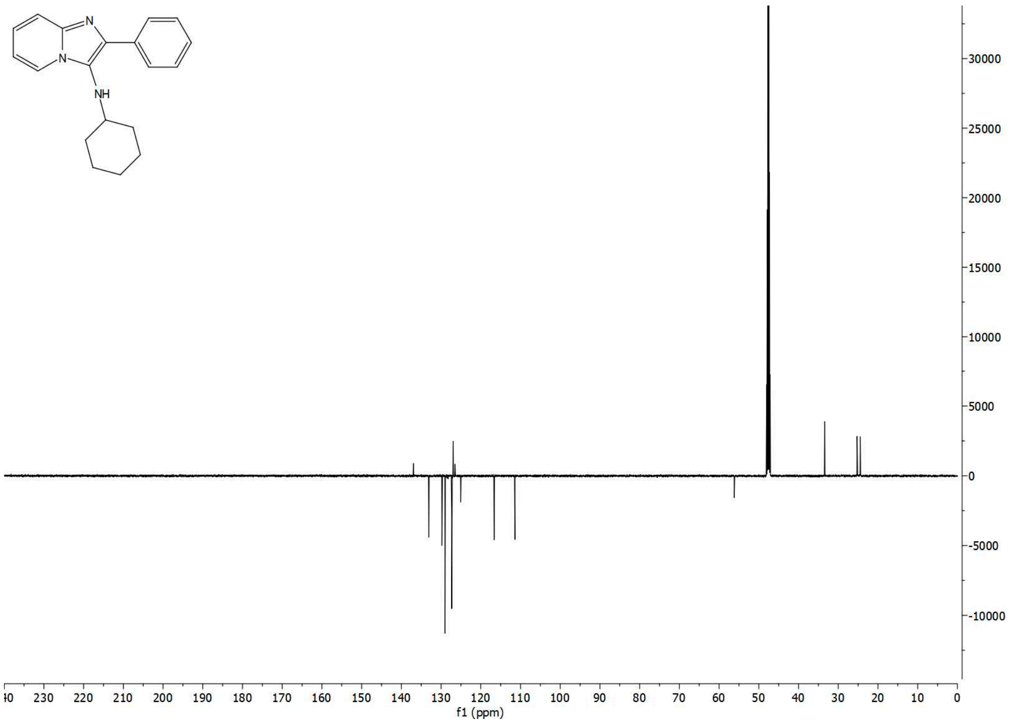

Figure S2. Compound 1 ^{13}C NMR (151 MHz, Methanol-d4)

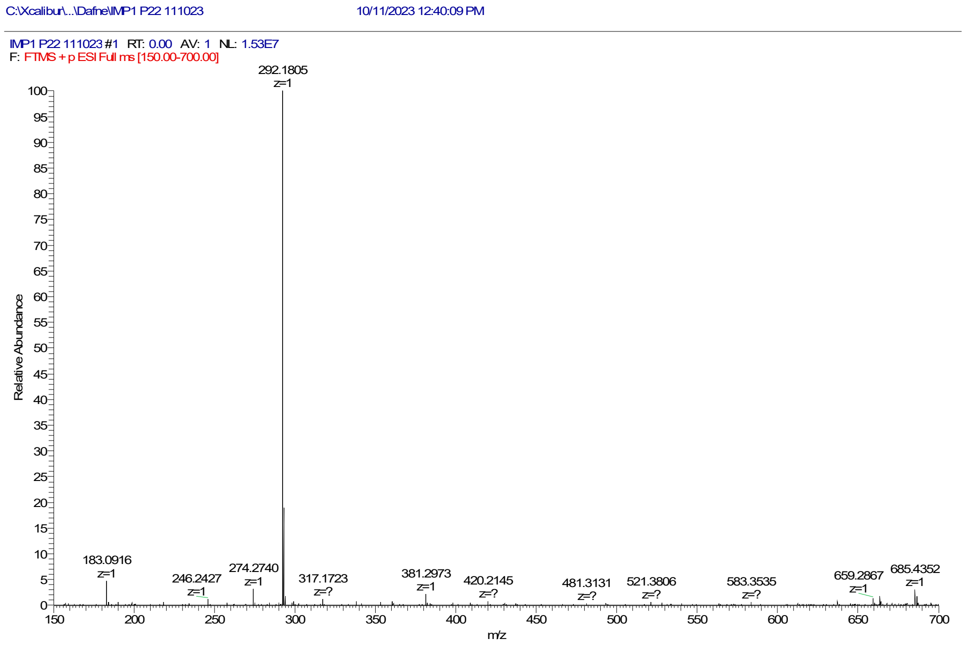

Figure S3. Compound 1 ESI-MS spectrum

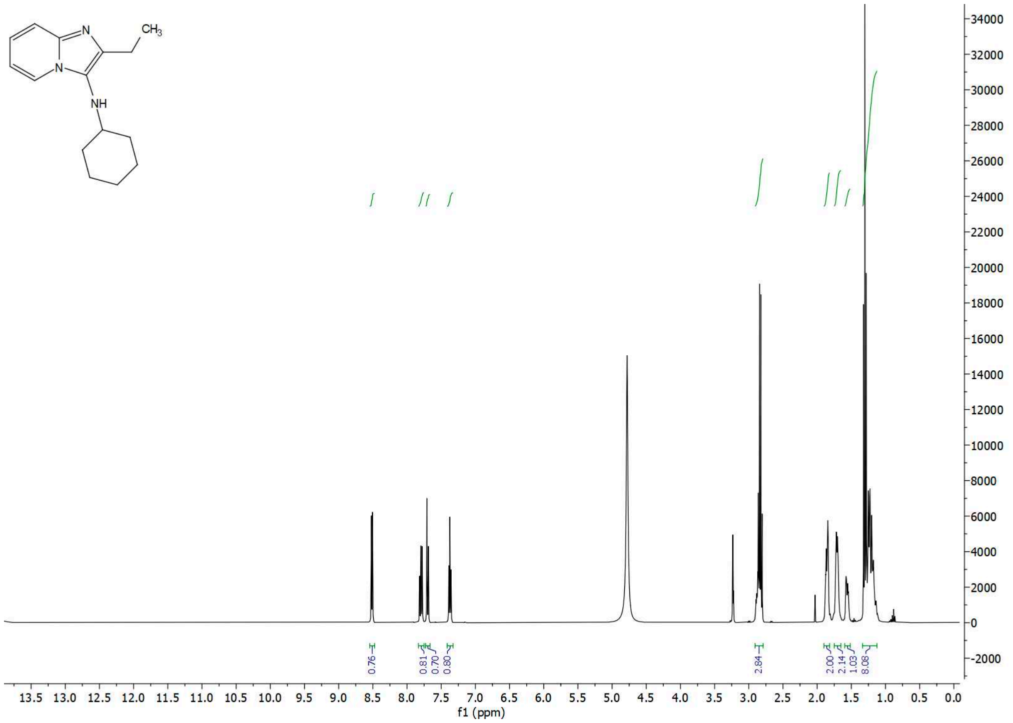

Figure S4. Compound 2 ^{1}H NMR (400 MHz, Methanol-d4)

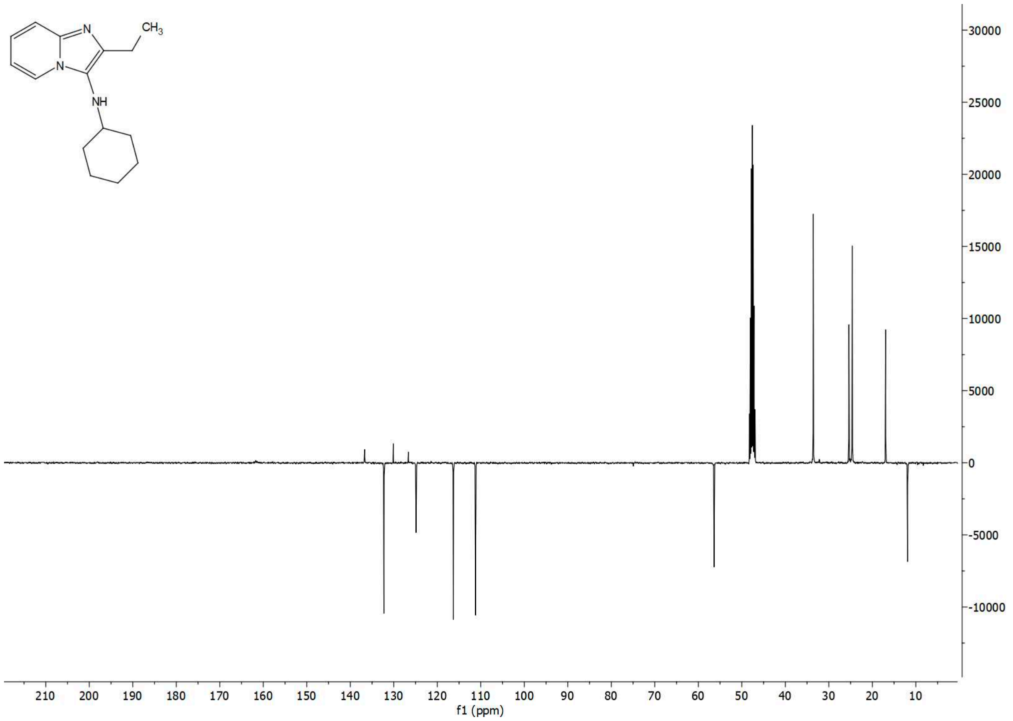

Figure S5. Compound 2 ^{13}C NMR (101 MHz, Methanol-d4)

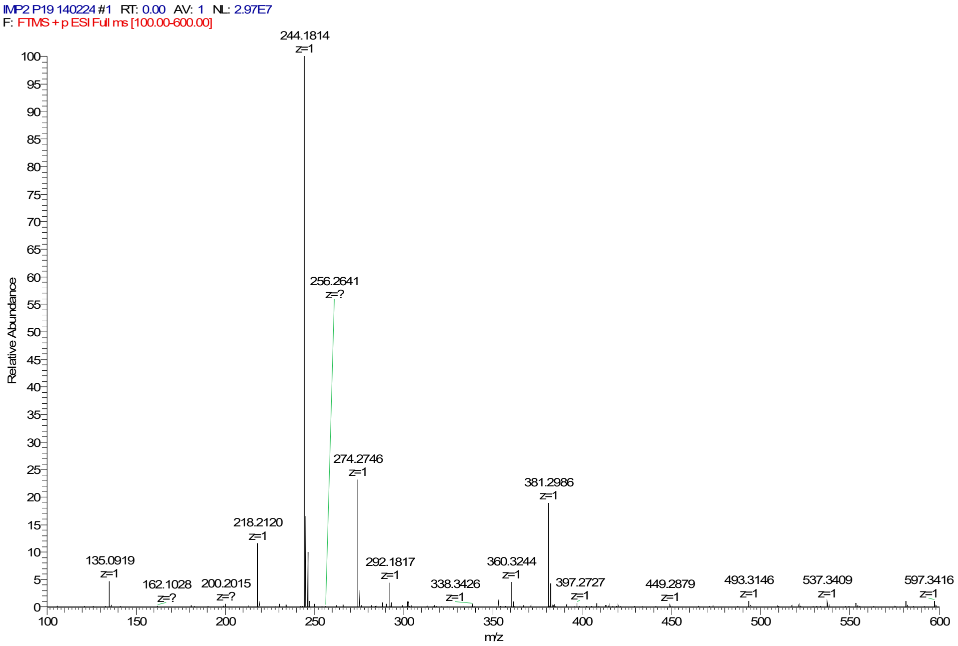

Figure S6. Compound 2 ESI-MS spectrum

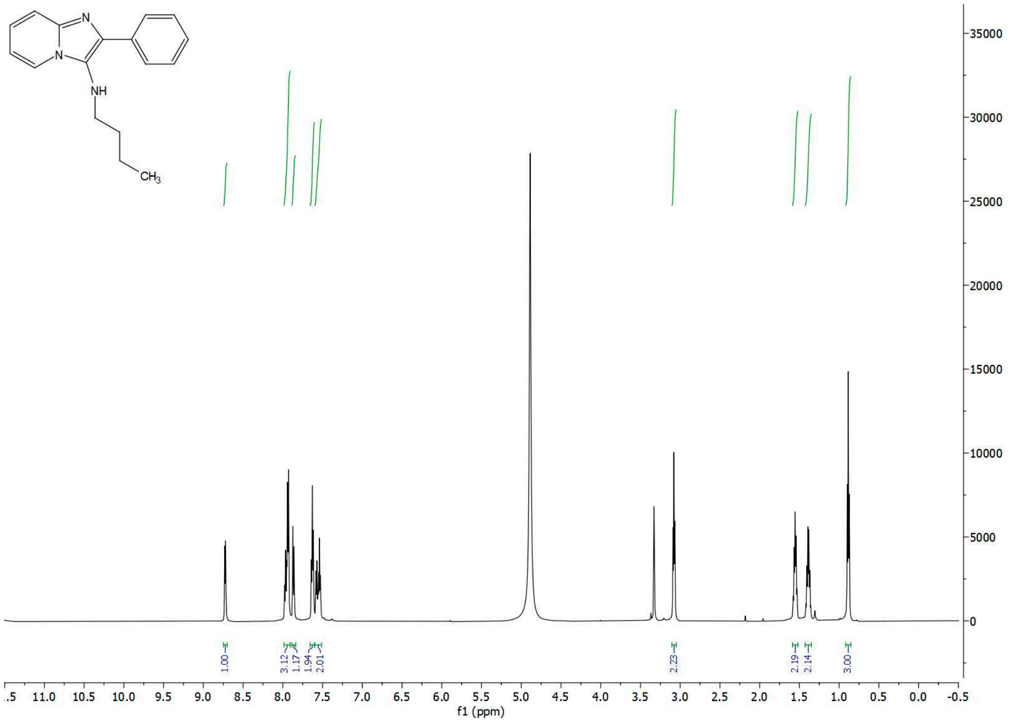

Figure S7. Compound 3 ^{1}H NMR (600 MHz, Methanol-d4)

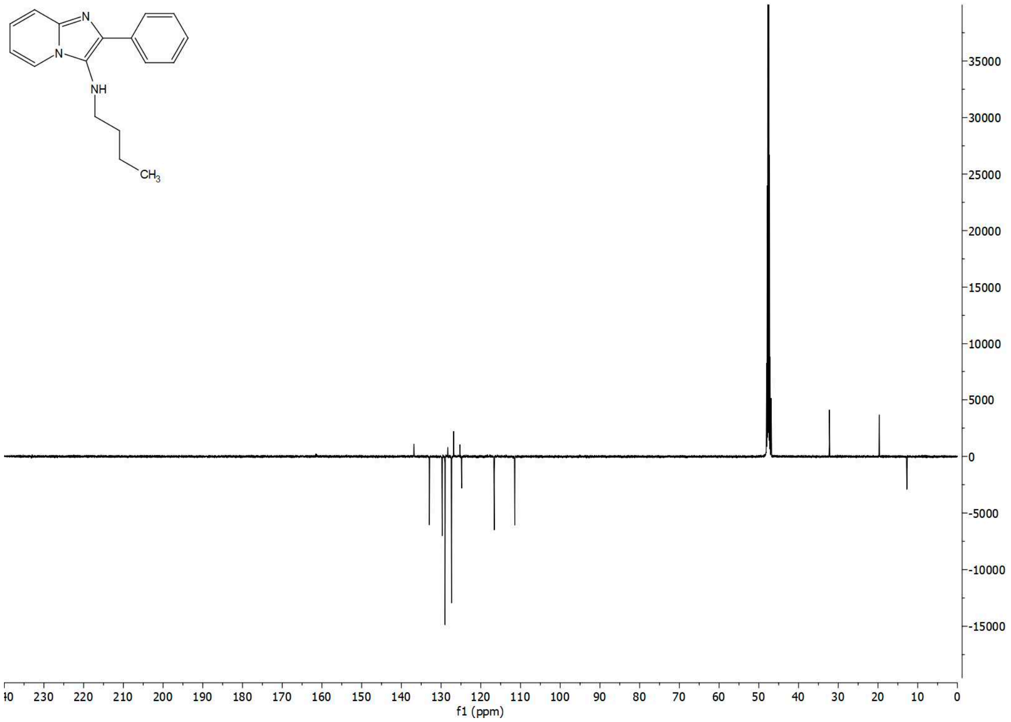

Figure S8. Compound 3 ^{13}C NMR (151 MHz, Methanol-d4)

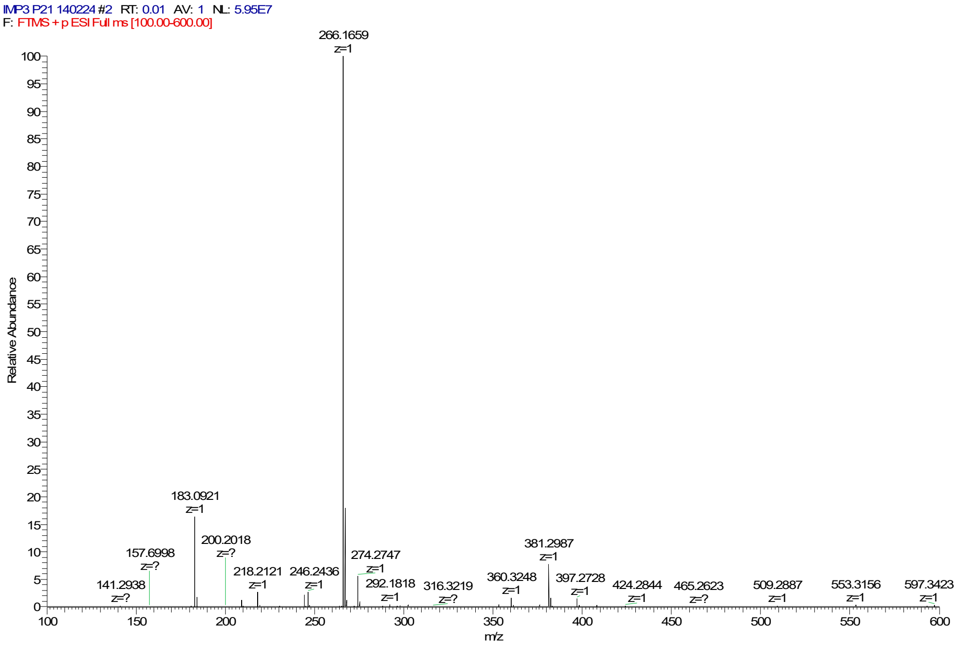

Figure S9. Compound 3 ESI-MS spectrum

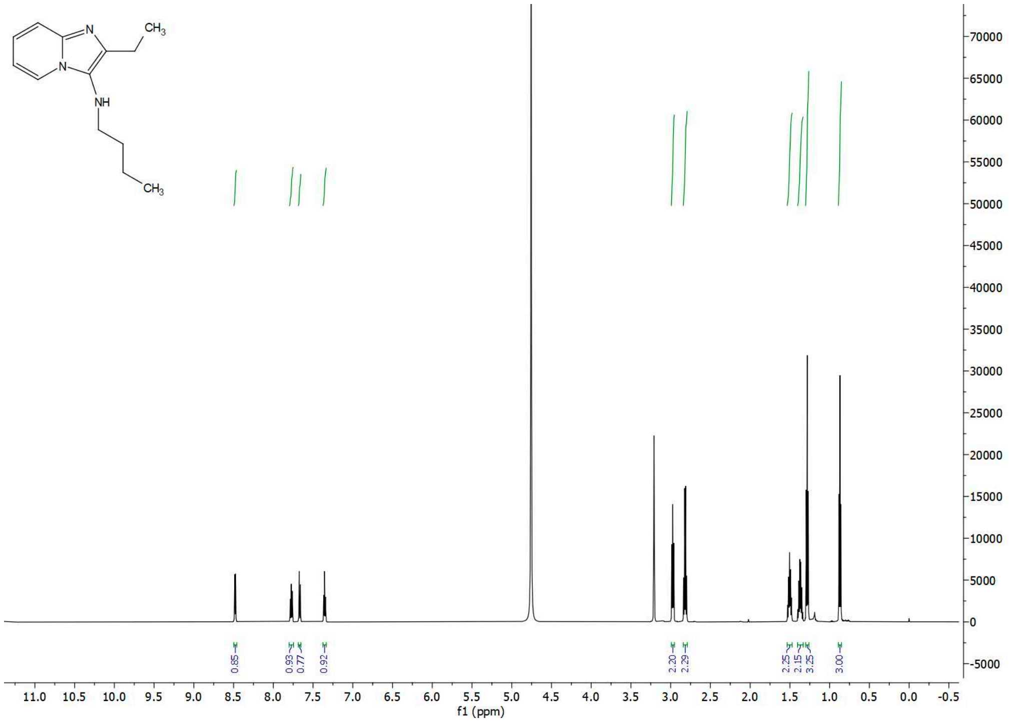

Figure S10. Compound 4 ^{1}H NMR (600 MHz, Methanol-d4)

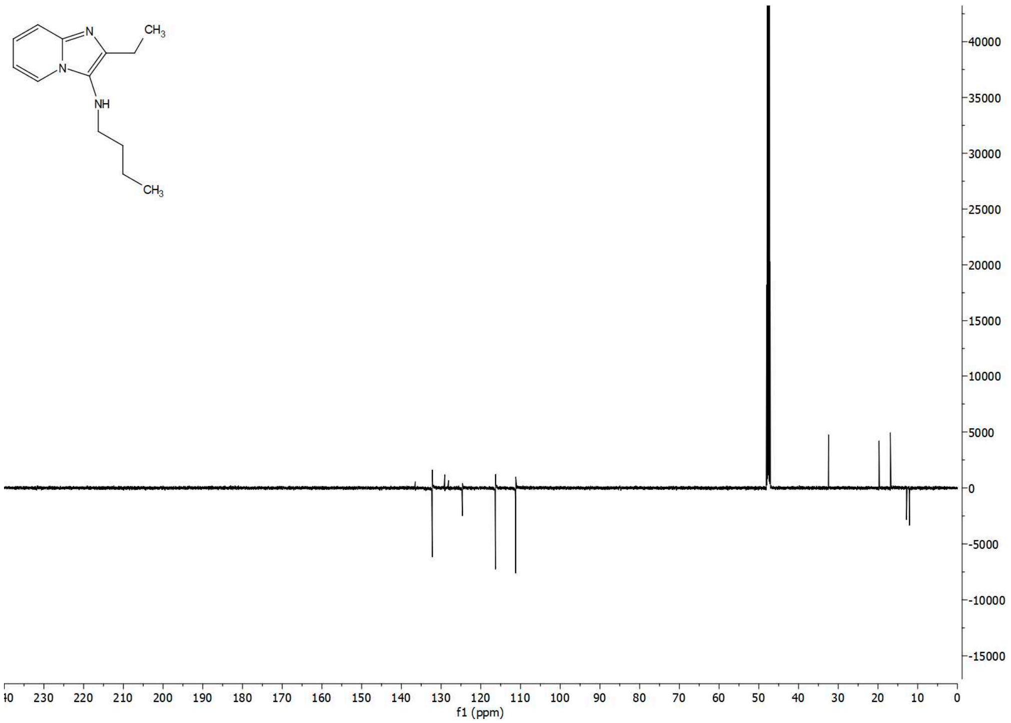

Figure S11. Compound 4 ^{13}C NMR (151 MHz, Methanol-d4)

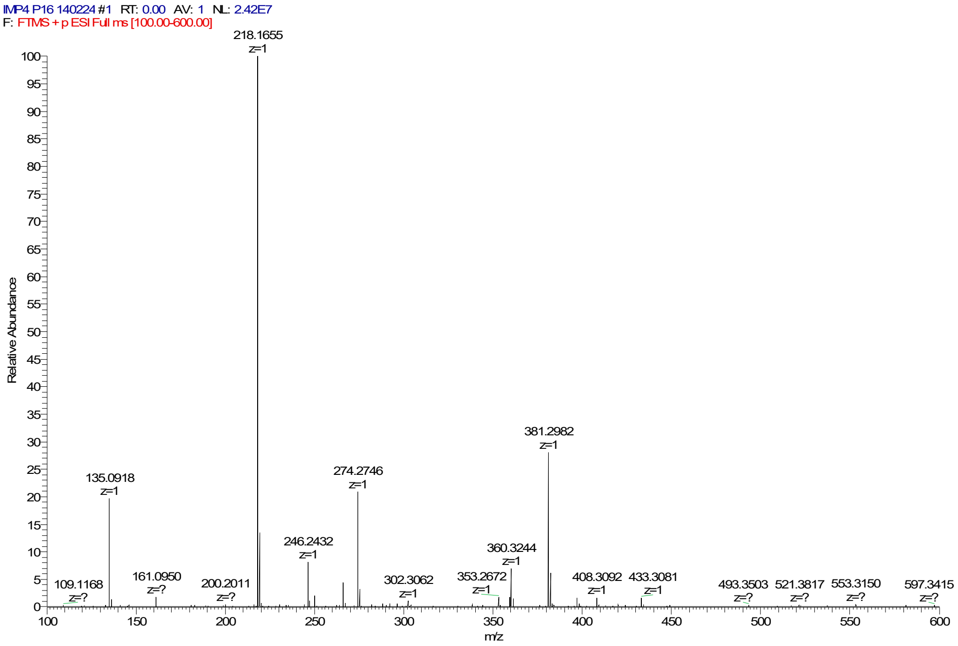

Figure S12. Compound 4 ESI-MS spectrum

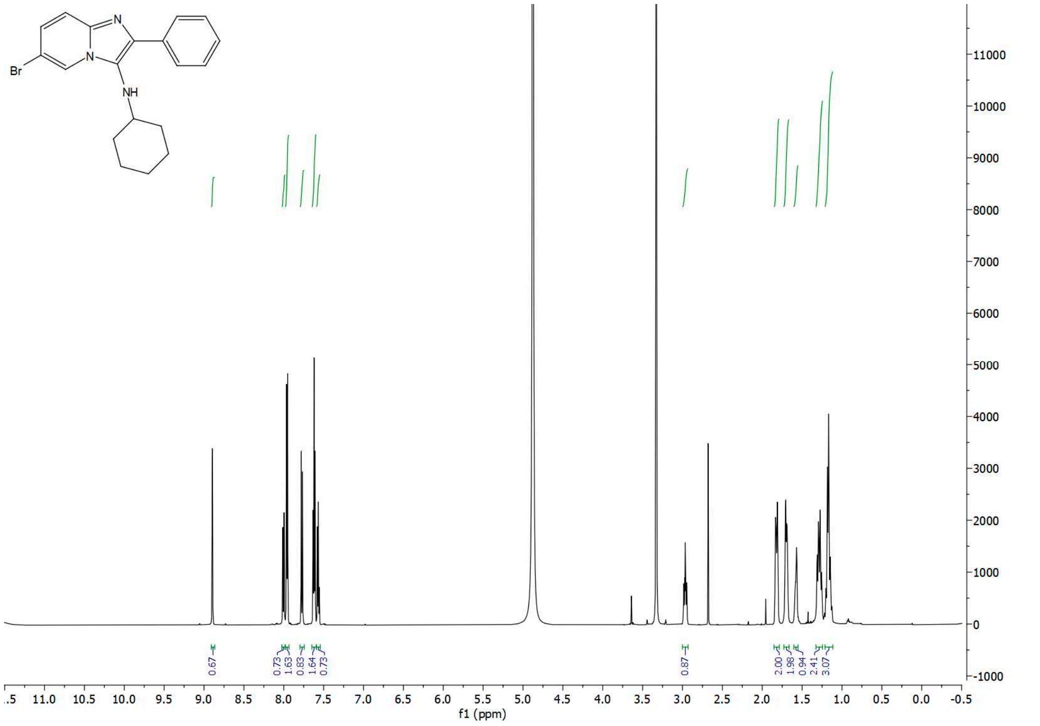

Figure S13. Compound 5 ^{1}H NMR (600 MHz, Methanol-d4)

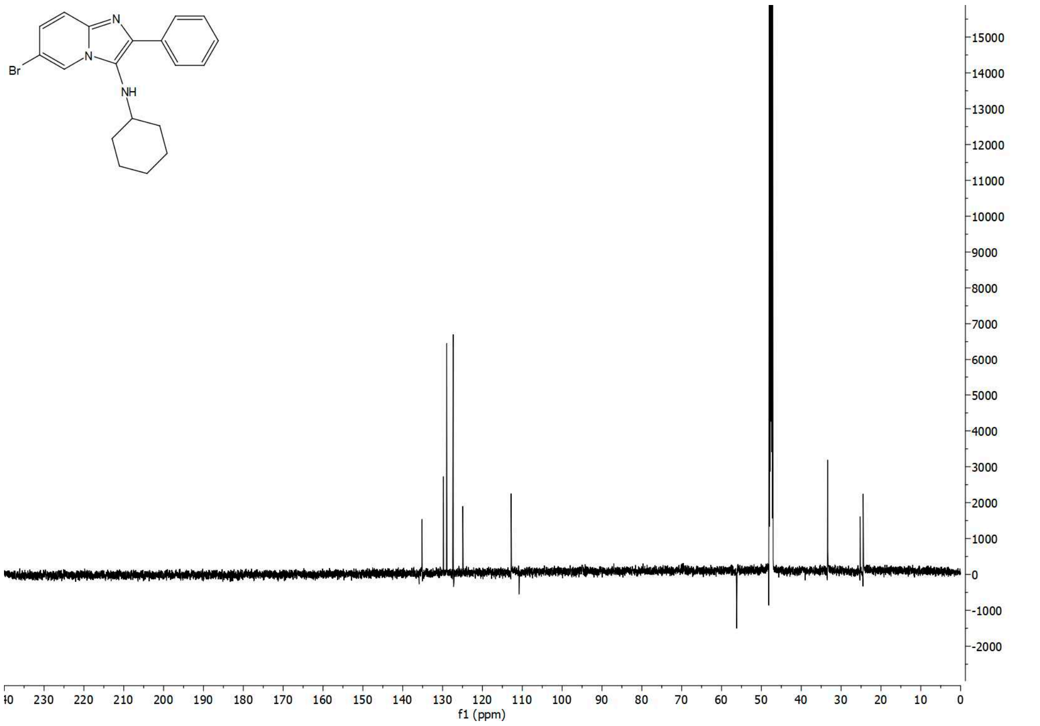

Figure S14. Compound 5 ^{13}C NMR (151 MHz, Methanol-d4)

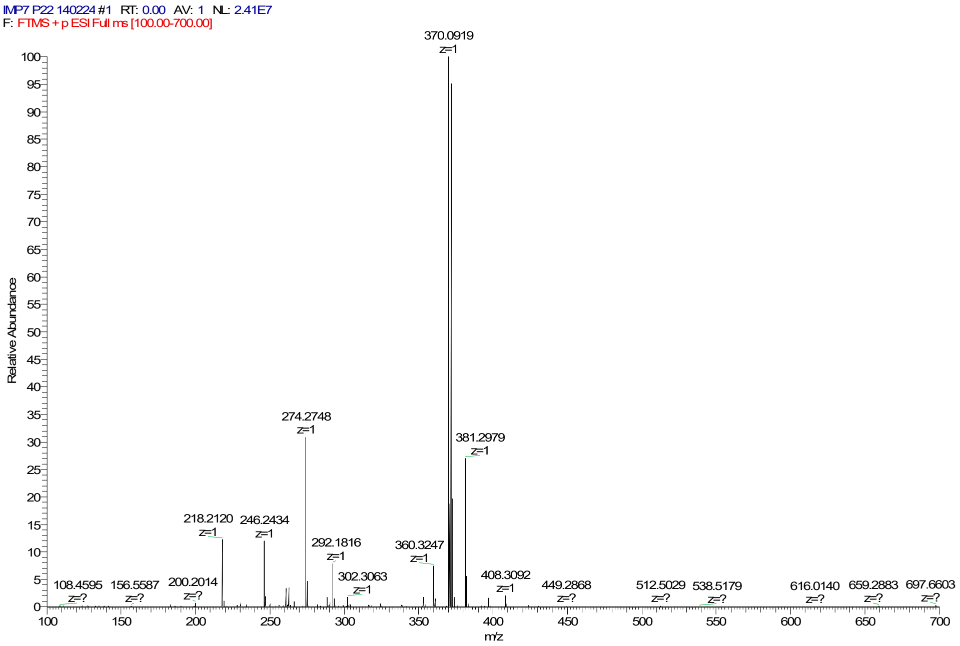

Figure S15. Compound 5 ESI-MS spectrum

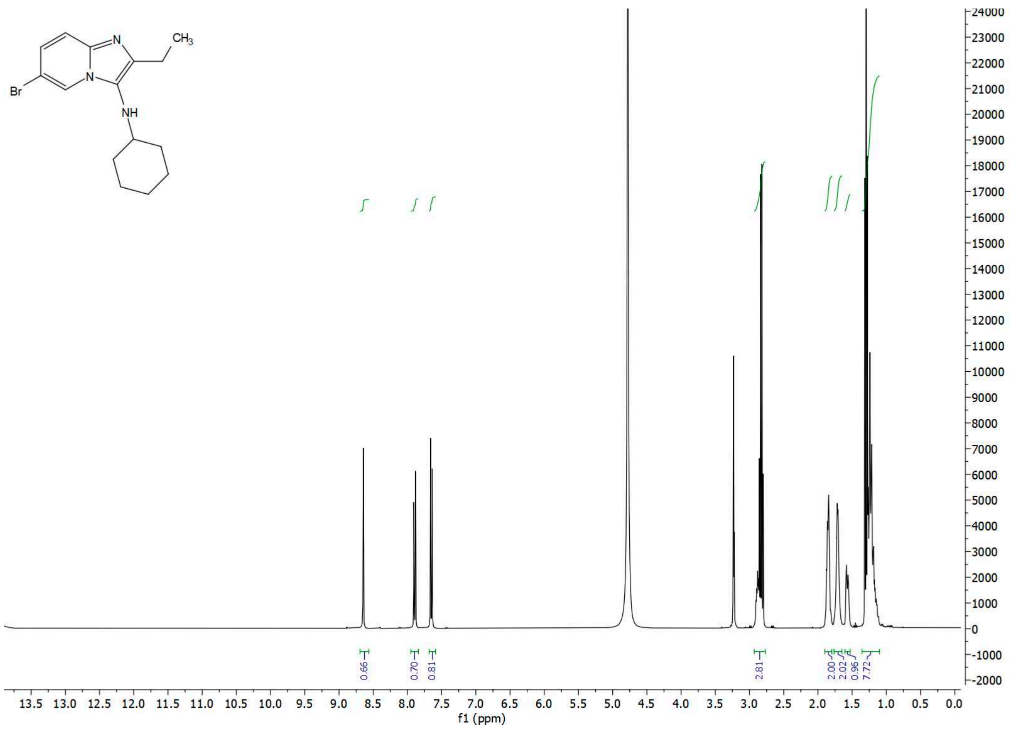

Figure S16. Compound 6 ^{1}H NMR (400 MHz, Methanol-d4)

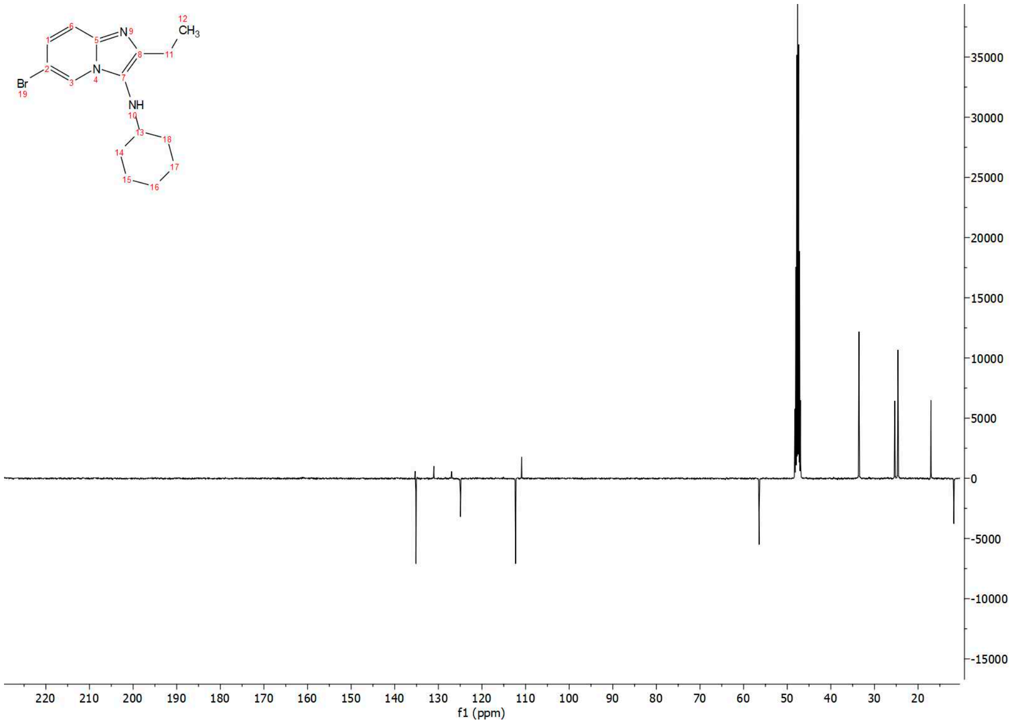

Figure S17. Compound 6 ^{13}C NMR (101 MHz, Methanol-d4)

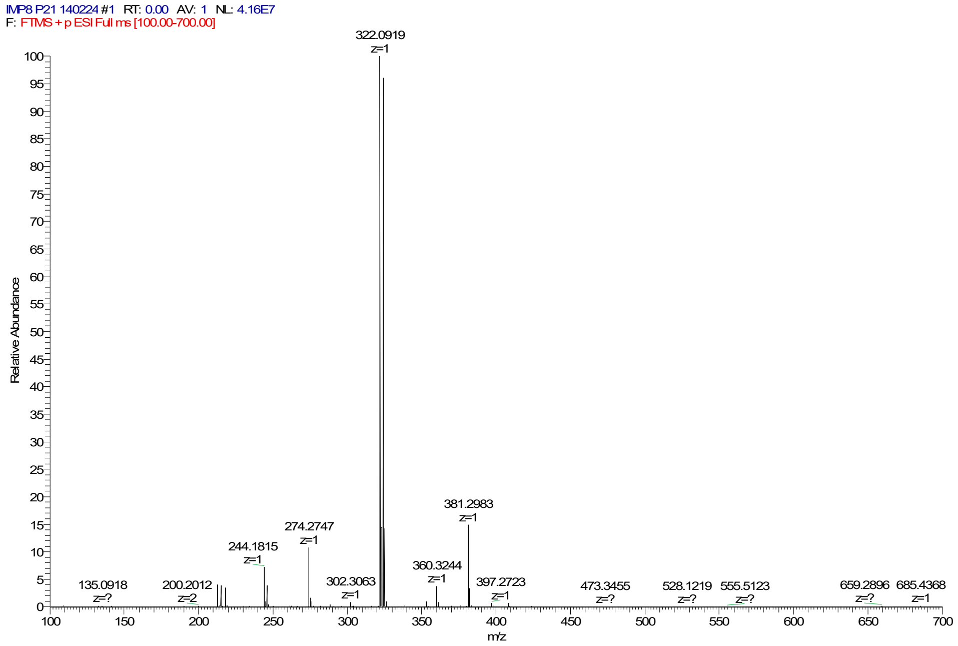

Figure S18. Compound 6 ESI-MS spectrum

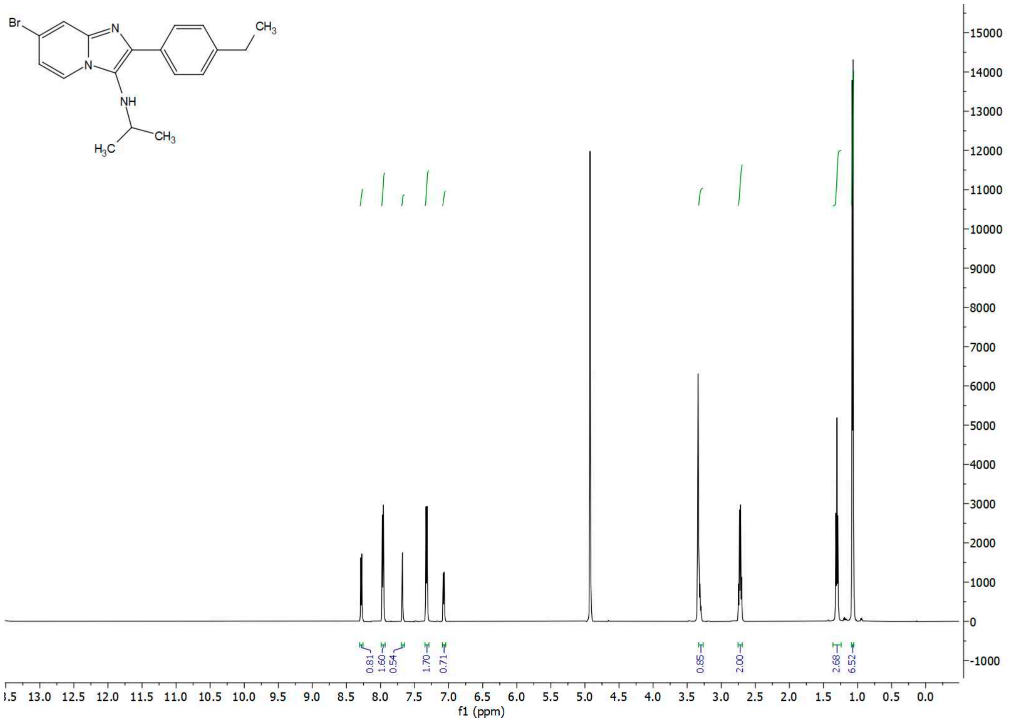

Figure S19. Compound 7 ^{1}H NMR (500 MHz, Methanol-d4)

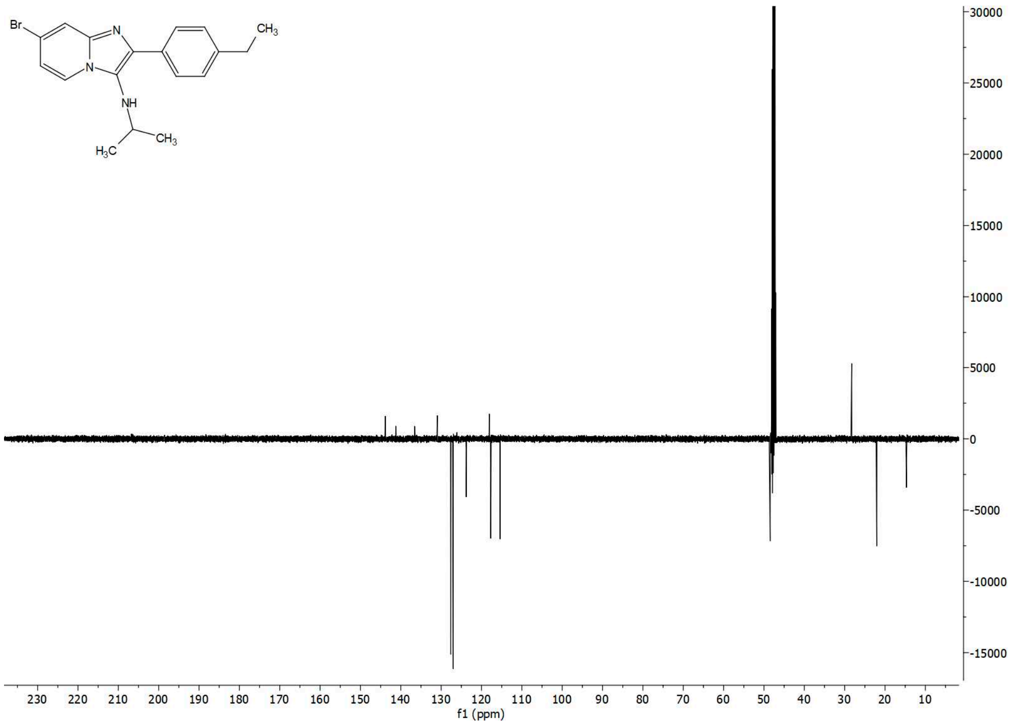

Figure S20. Compound 7 ^{13}C NMR (126 MHz, Methanol-d4)

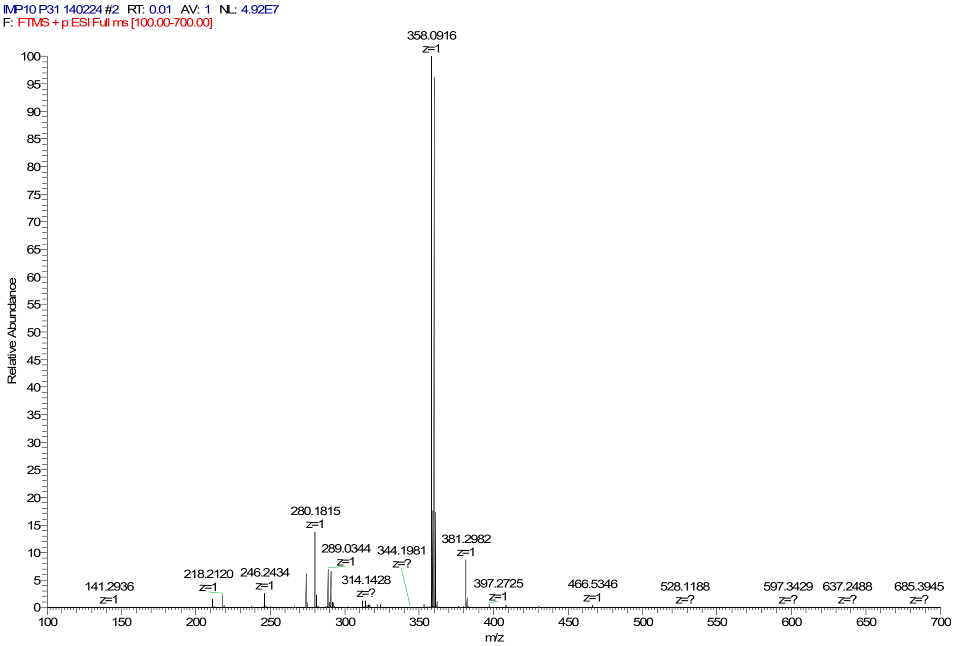

Figure S21. Compound 7 ESI-MS spectrum

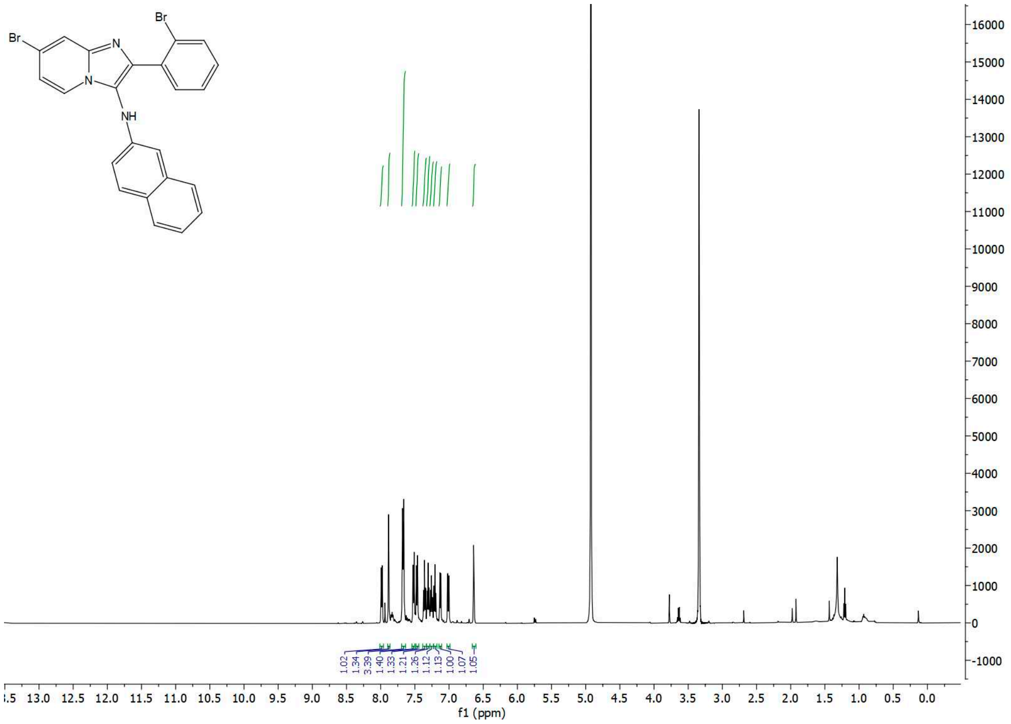

Figure S22. Compound 8 ^{1}H NMR (500 MHz, Methanol-d4)

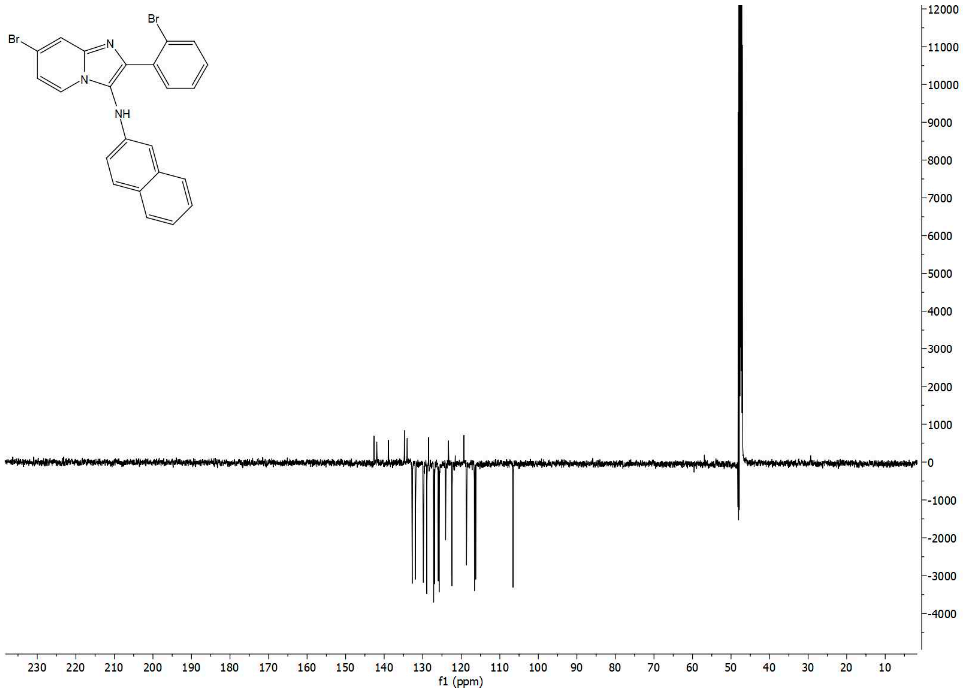

Figure S23. Compound 8 ^{13}C NMR (126 MHz, Methanol-d4)

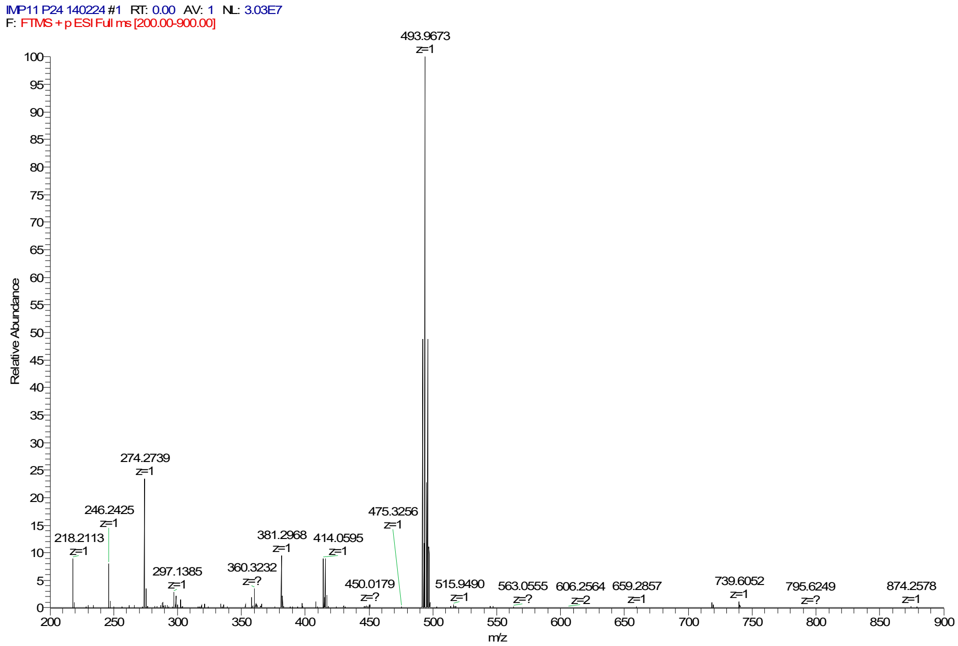

Figure S24. Compound 8 ESI-MS spectrum

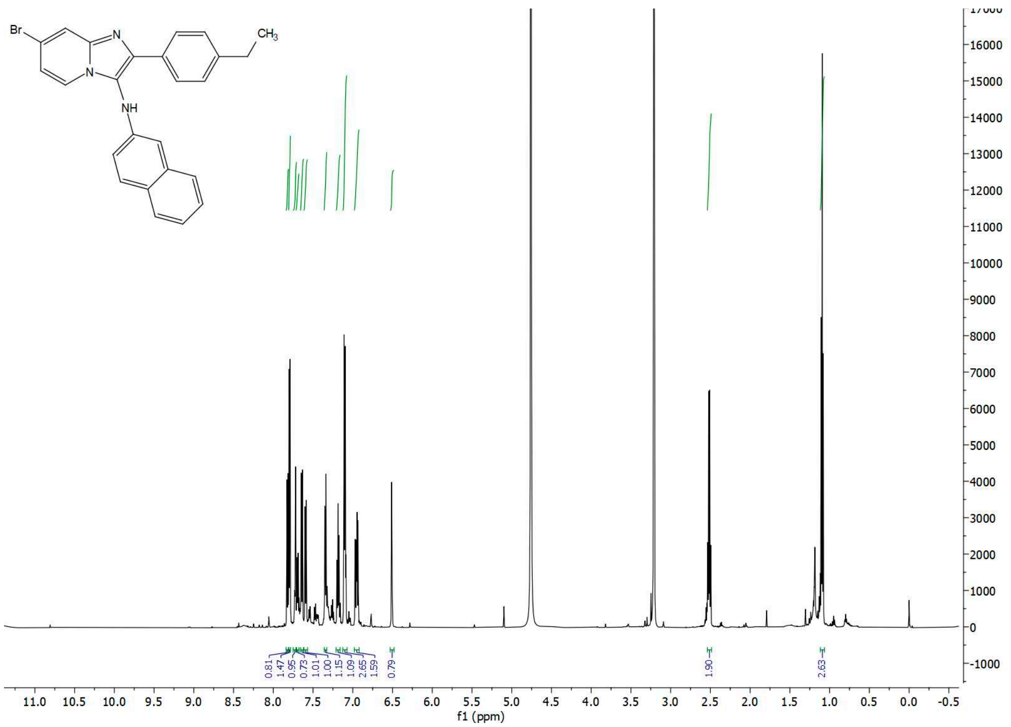

Figure S25. Compound 9 ^{1}H NMR (600 MHz, Methanol-d4)

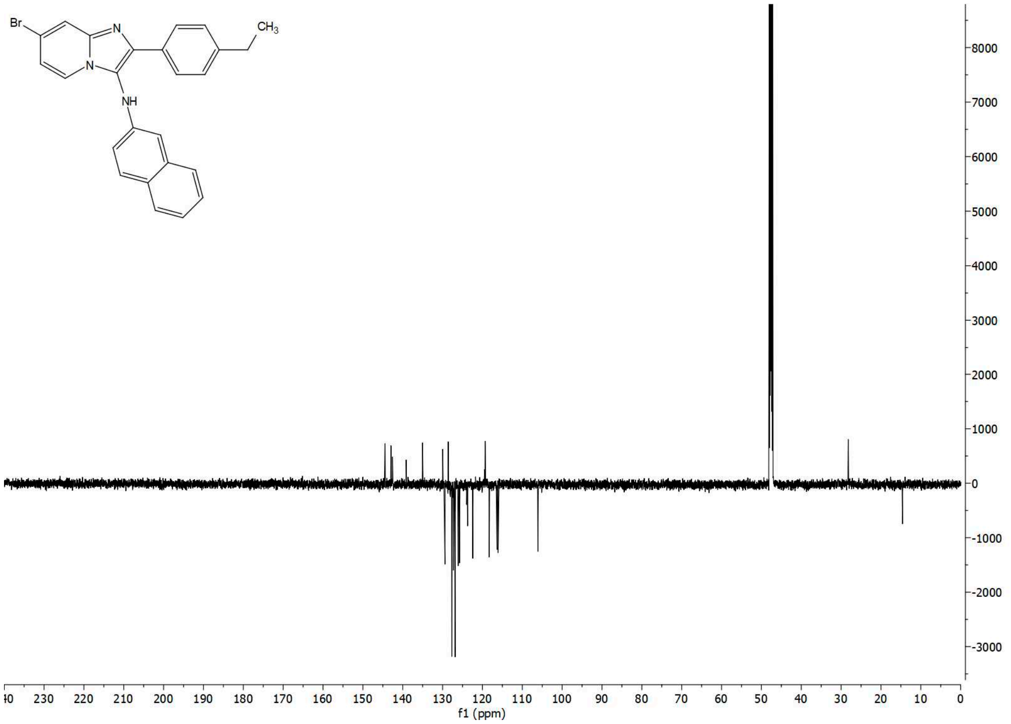

Figure S26. Compound 9 ^{13}C NMR (151 MHz, Methanol-d4)

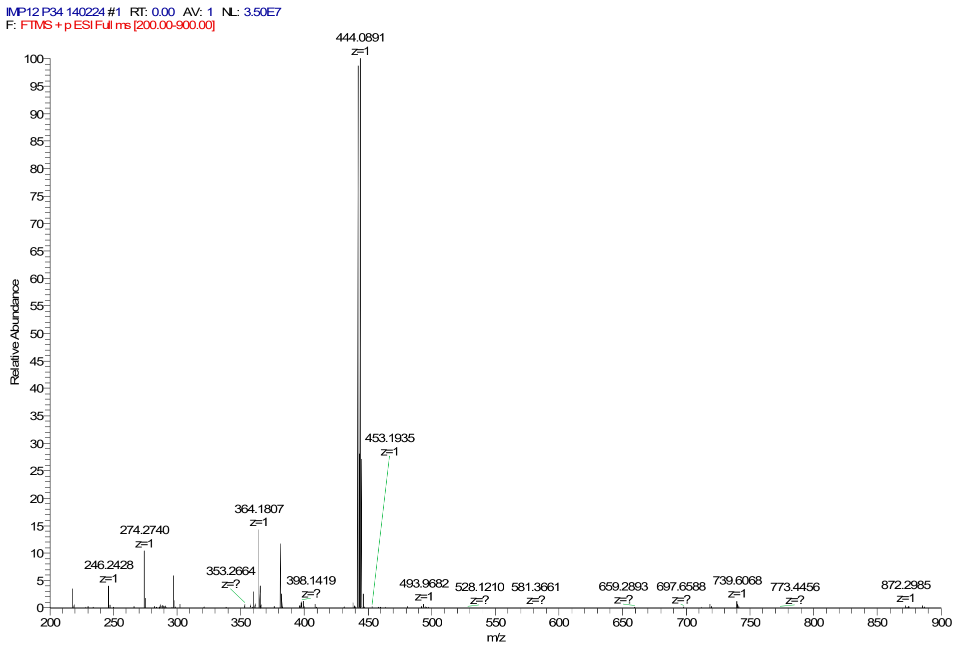

Figure S27. Compound 9 ESI-MS spectrum

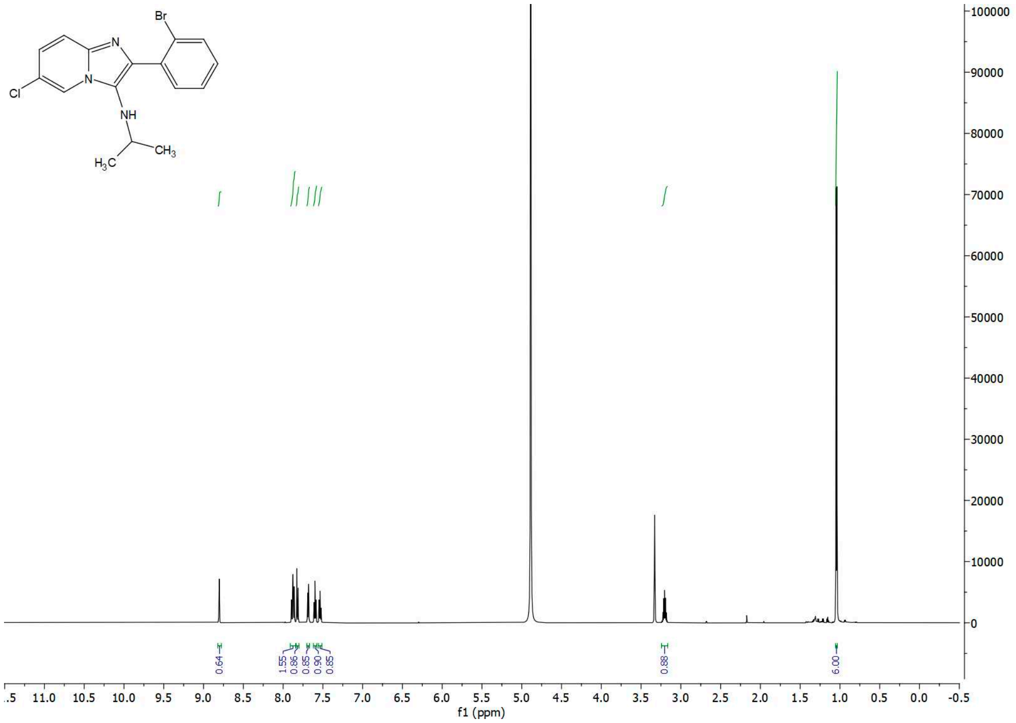

Figure S28. Compound 10 ^{1}H NMR (600 MHz, Methanol-d4)

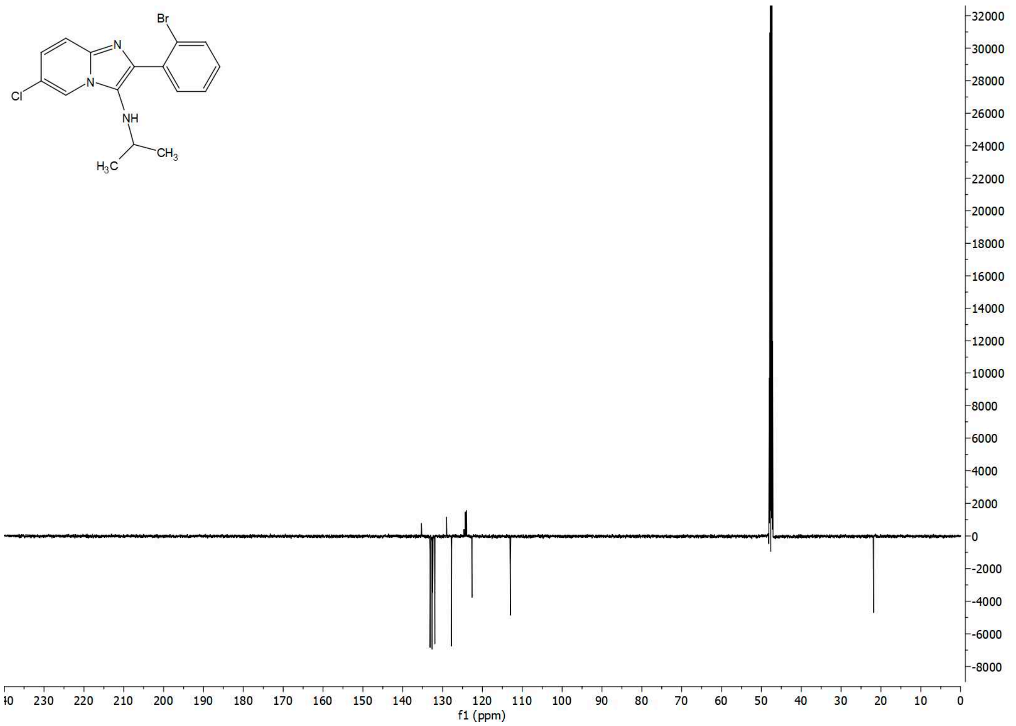

Figure S29. Compound 10 ^{13}C NMR (151 MHz, Methanol-d4)

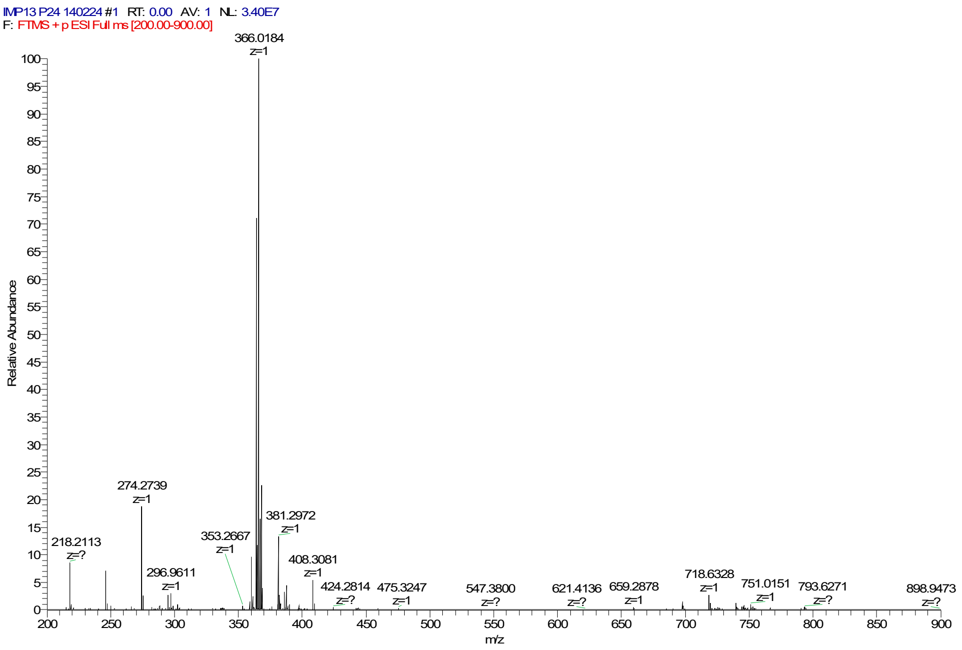

Figure S30. Compound 10 ESI-MS spectrum

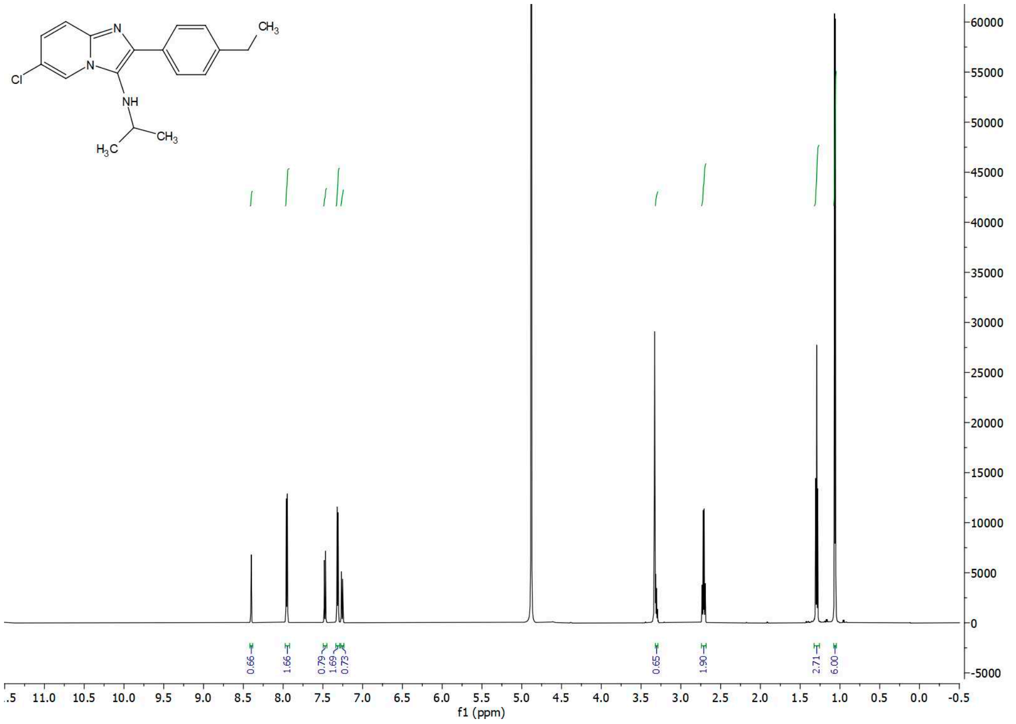

Figure S31. Compound 11 ^{1}H NMR (600 MHz, Methanol-d4)

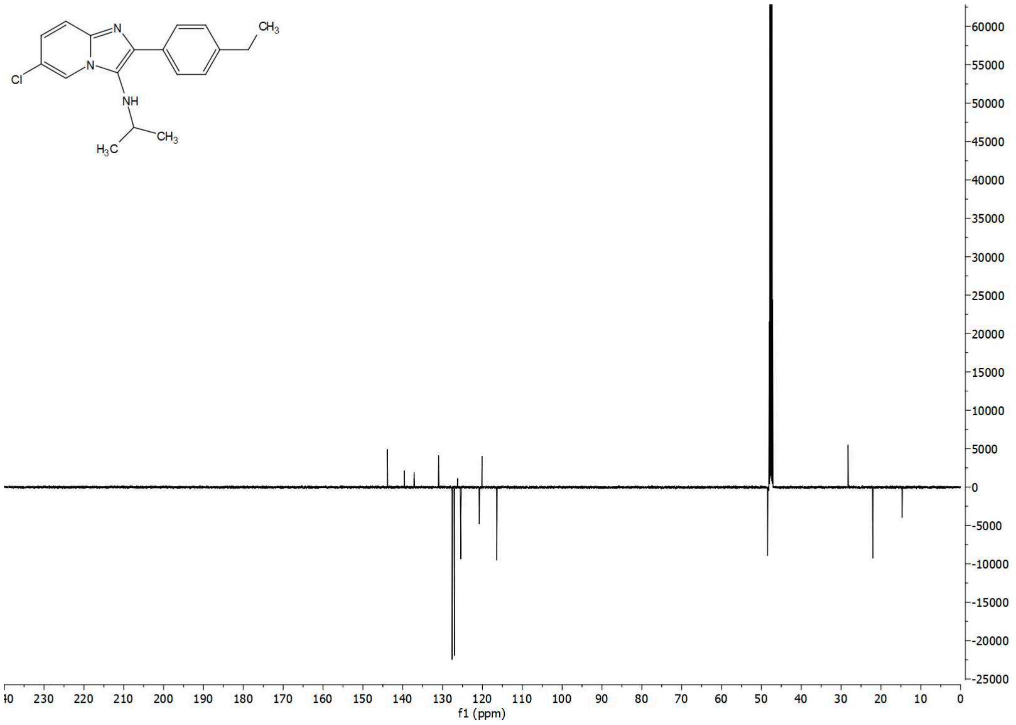

Figure S32. Compound 11 ^{13}C NMR (151 MHz, Methanol-d4)

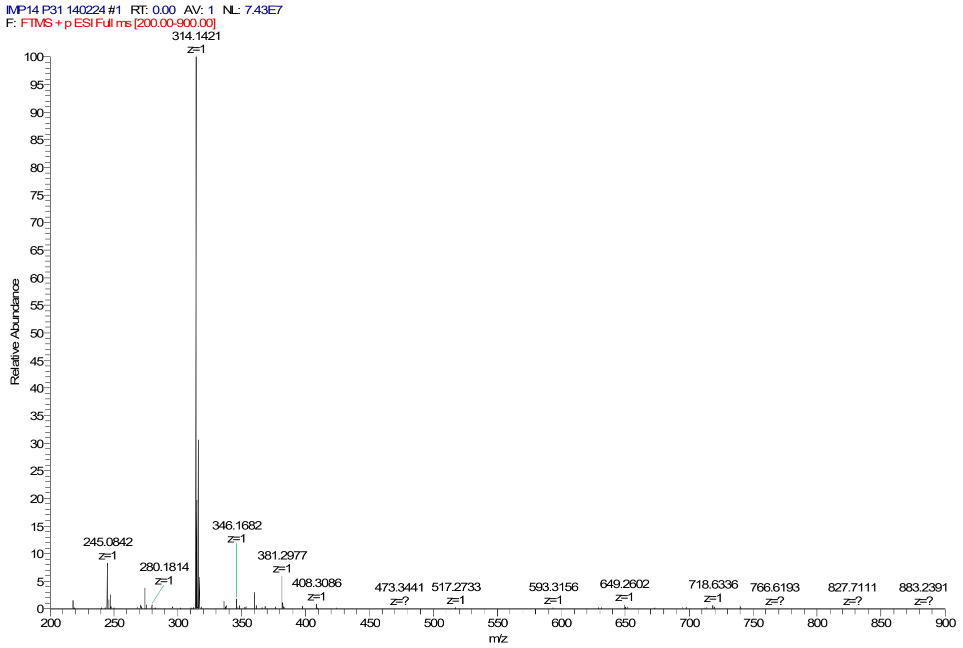

Figure S33. Compound 11 ESI-MS spectrum

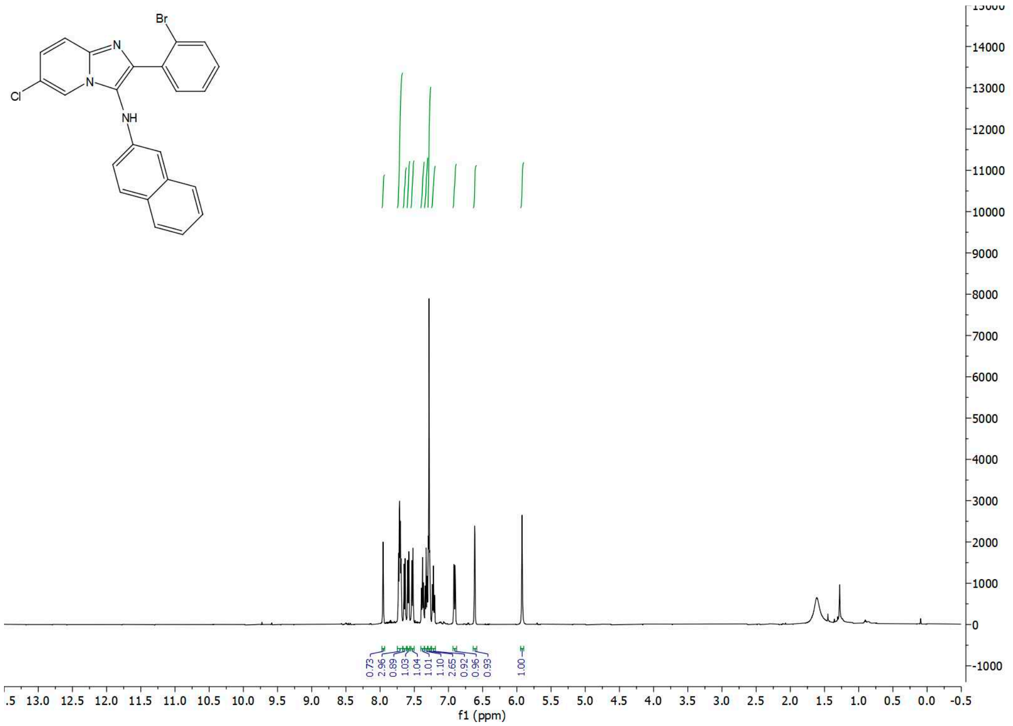

Figure S34. Compound 12 ^{1}H NMR (500 MHz, Chloroform-d)

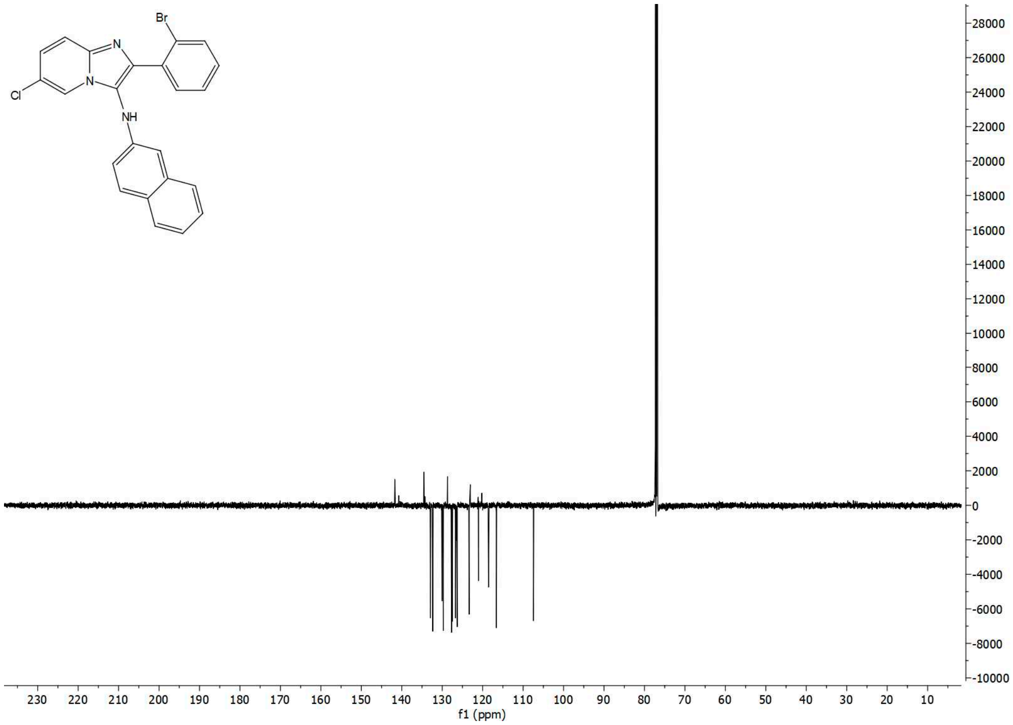

Figure S35. Compound 12 ^{13}C NMR (126 MHz, Chloroform-d)

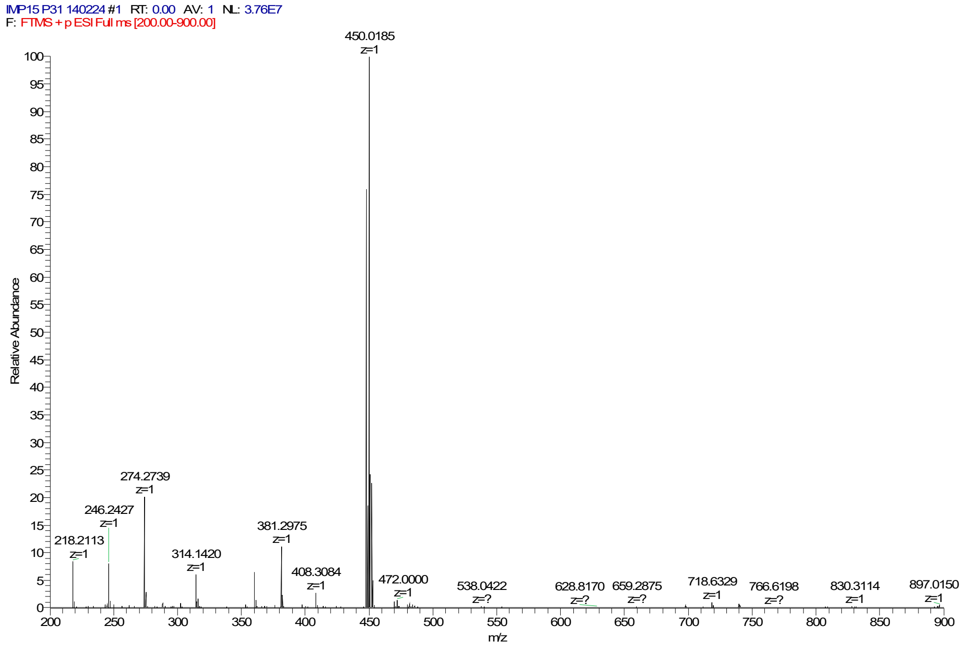

Figure S36. Compound 12 ESI-MS spectrum

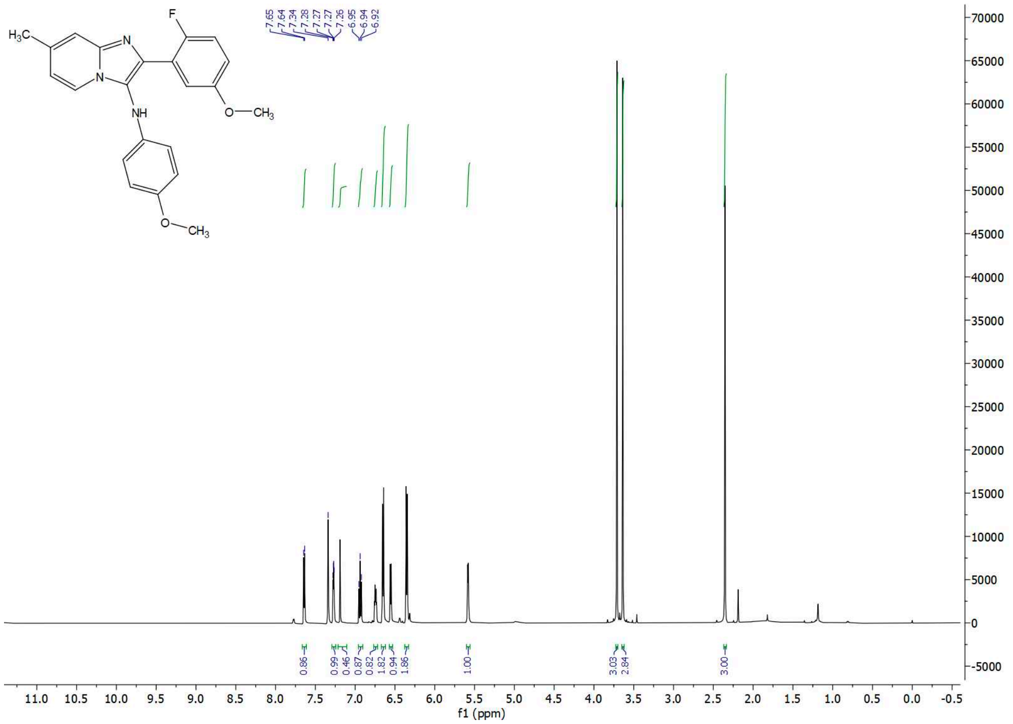

Figure S37. Compound 13 ^{1}H NMR (600 MHz, Chloroform-d)

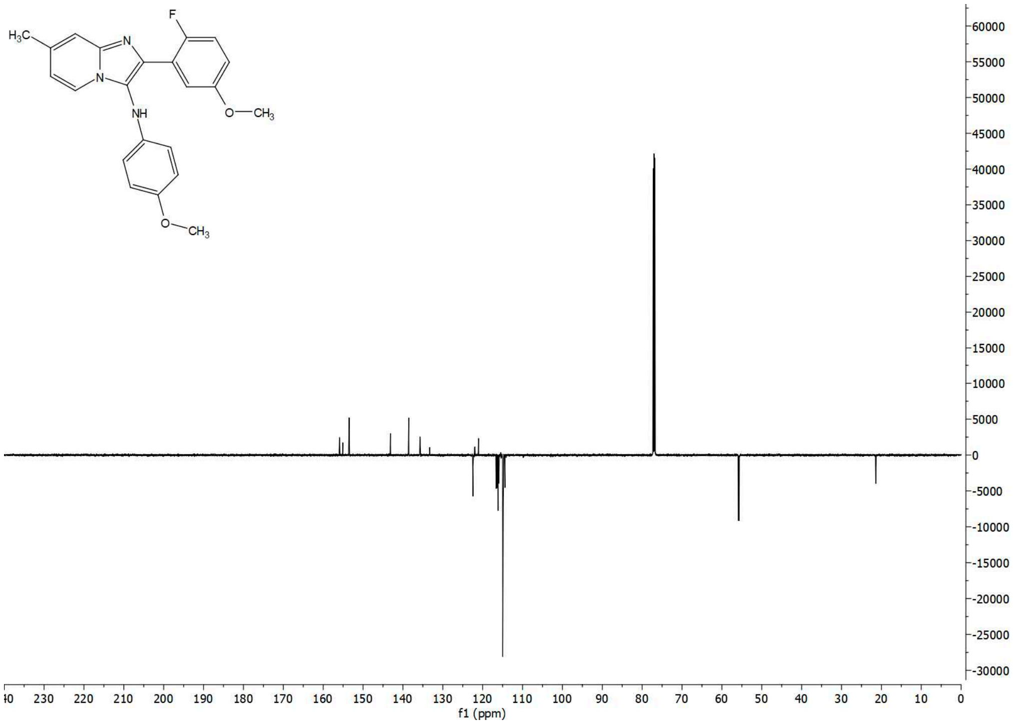

Figure S38. Compound 13 ^{13}C NMR (151 MHz, Chloroform-d)

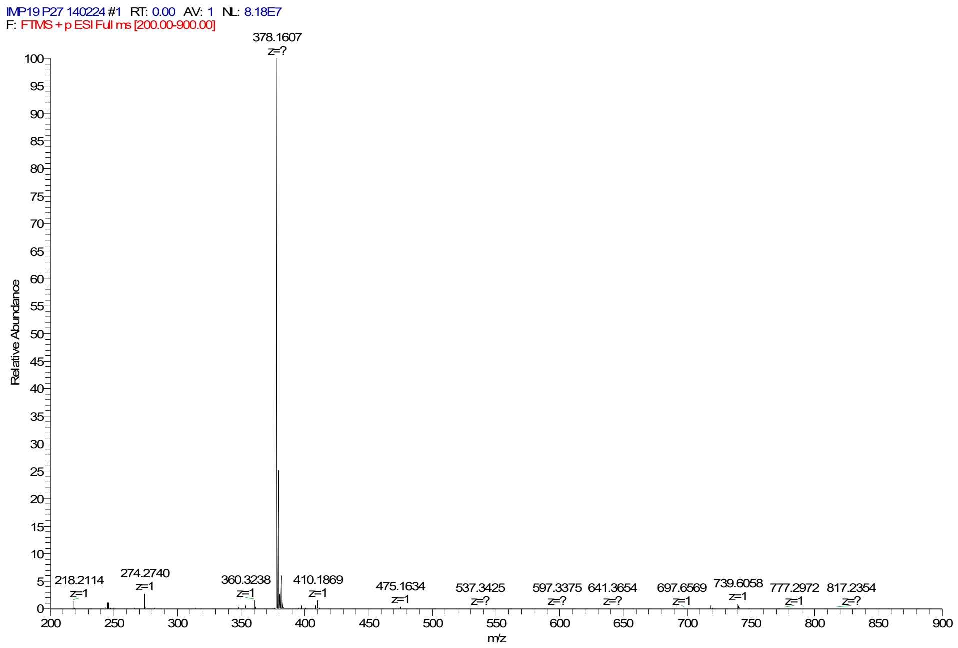

Figure S39. Compound 13 ESI-MS spectrum

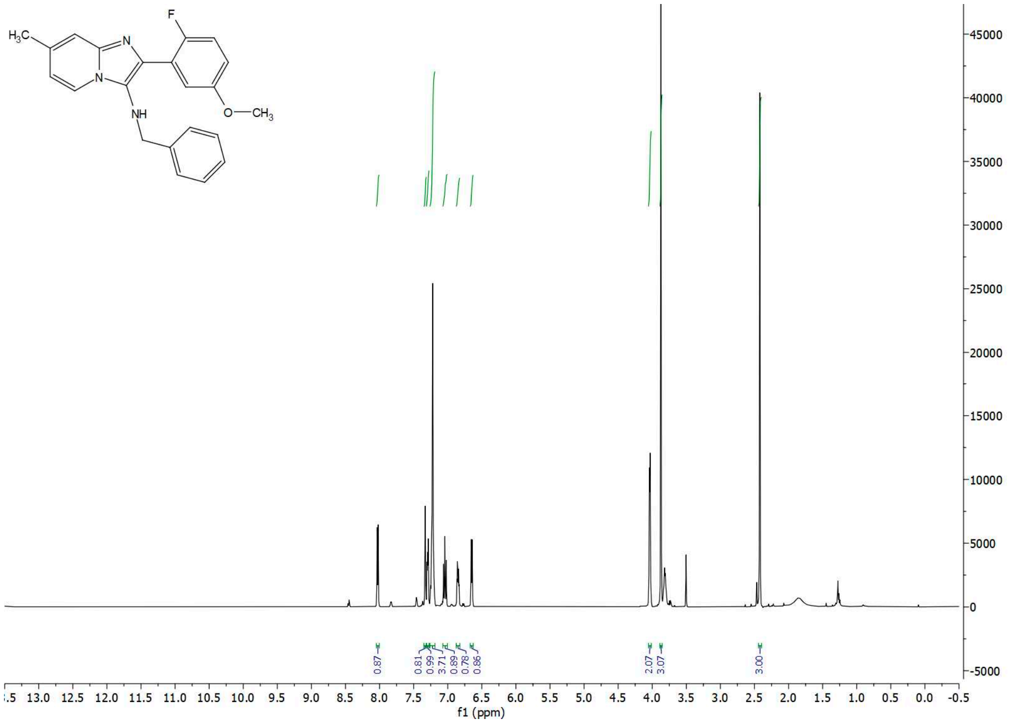

Figure S40. Compound 14 ^{1}H NMR (500 MHz, Chloroform-d)

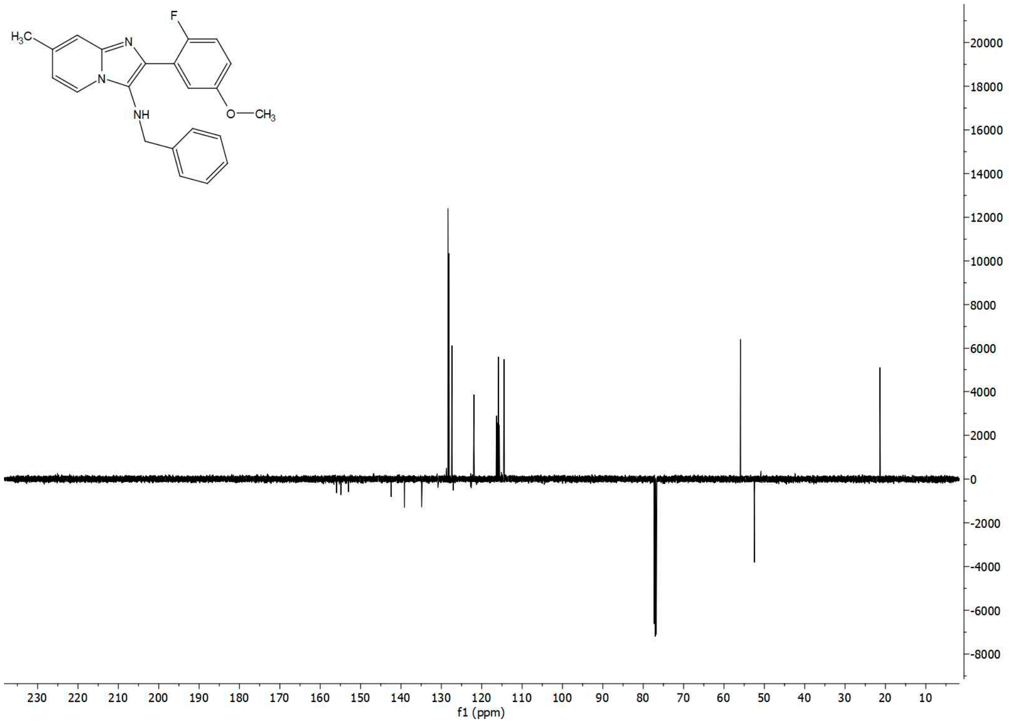

Figure S41. Compound 14 ^{13}C NMR (126 MHz, Chloroform-d)

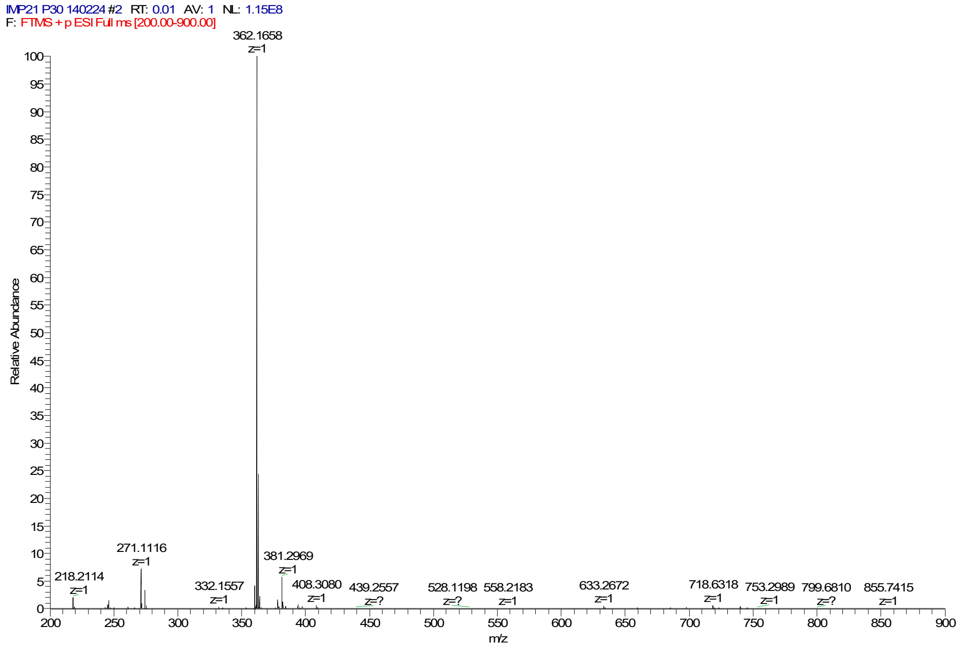

Figure S42. Compound 14 ESI-MS spectrum

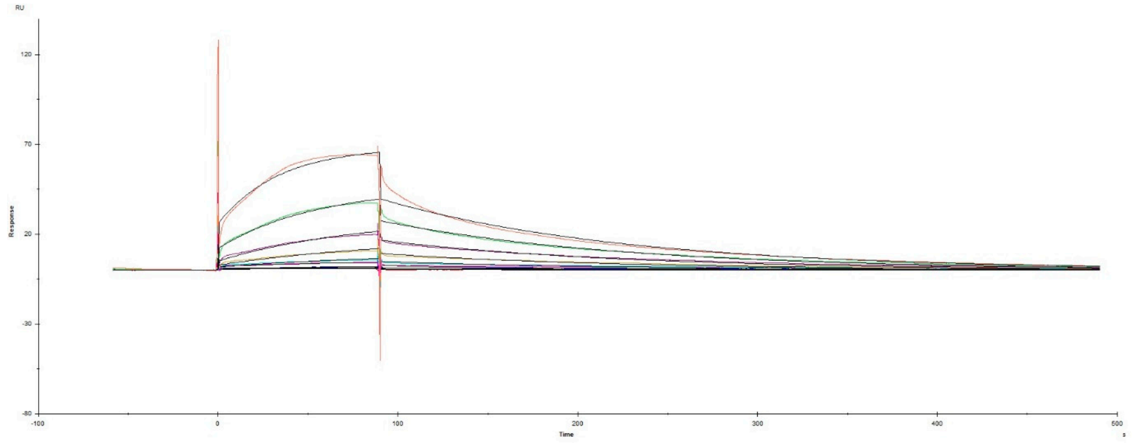

Figure S43. Sensorgram of compound 10 on BAG3 full length protein

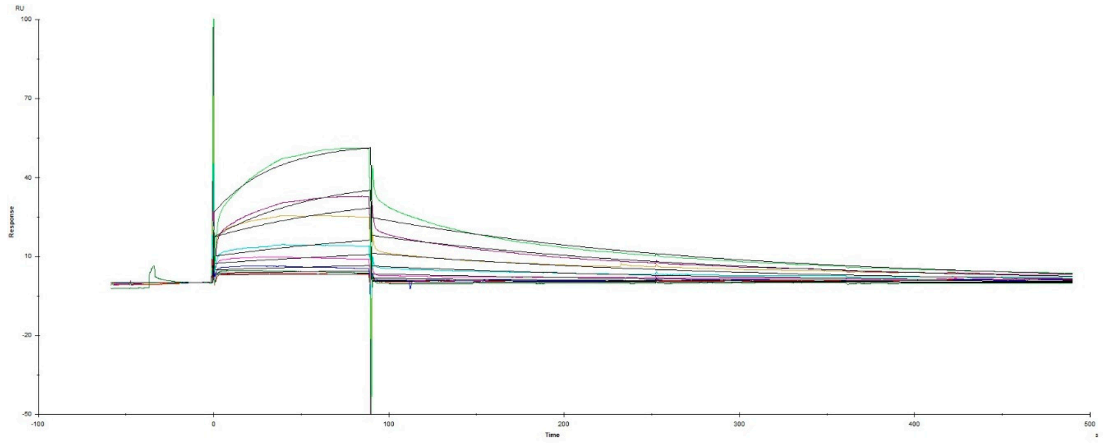

Figure S44. Sensorgram of compound 12 on BAG3 full length protein

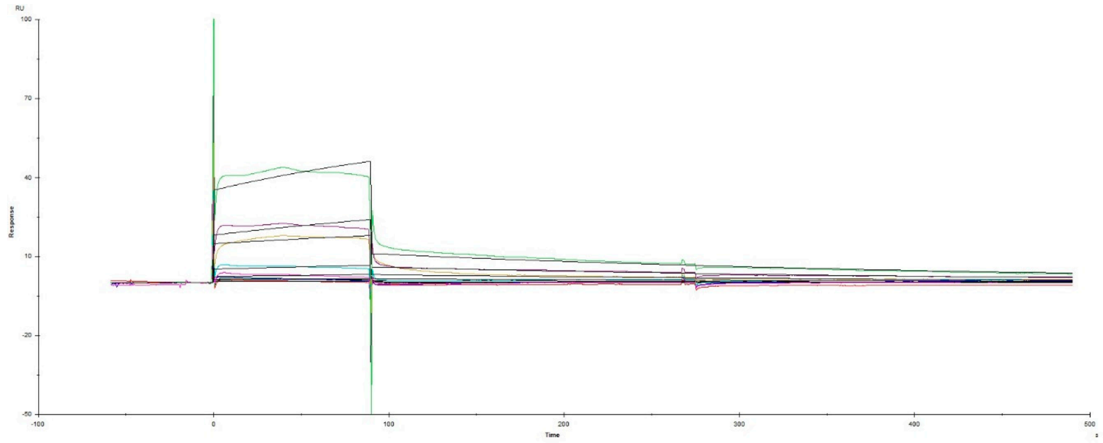

Figure S45. Sensorgram of compound 14 on BAG3 full length protein

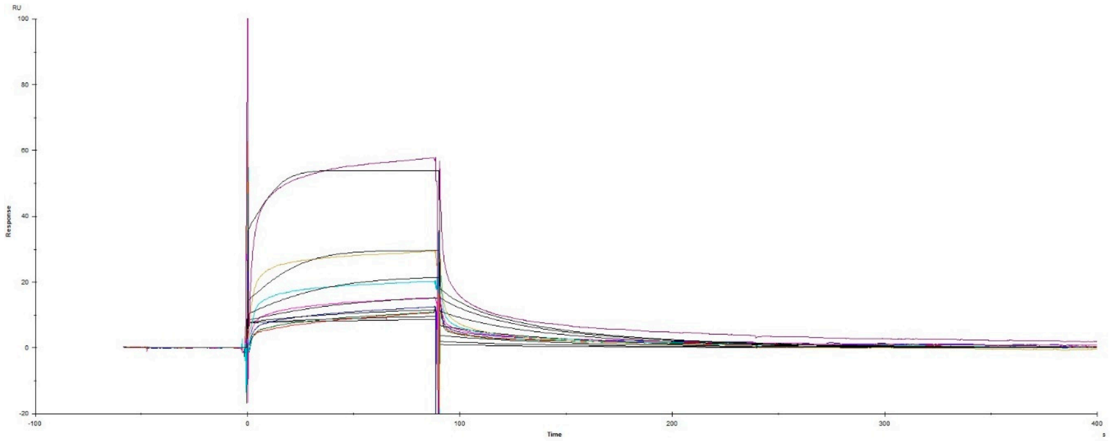

Figure S46. Sensorgram of LK4 on BAG3 full length protein

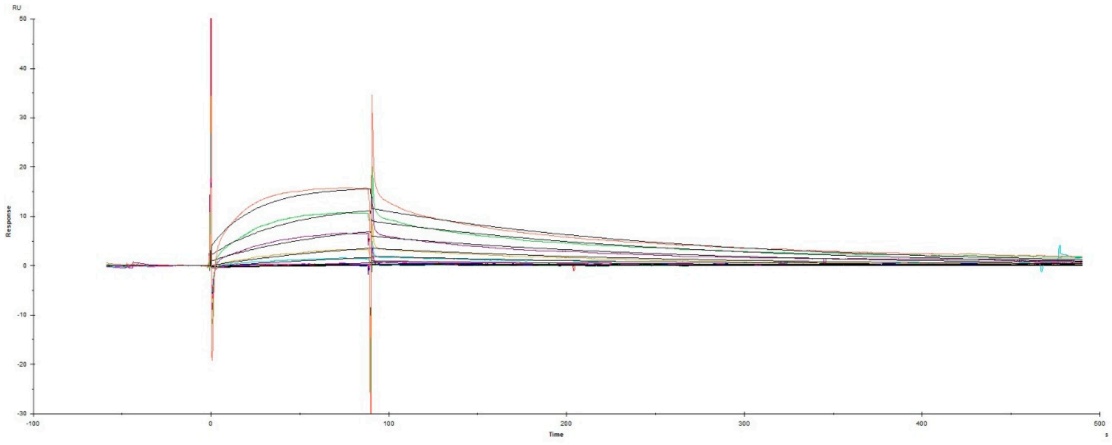

Figure S47. Sensorgram of compound 10 on BAG3-BD

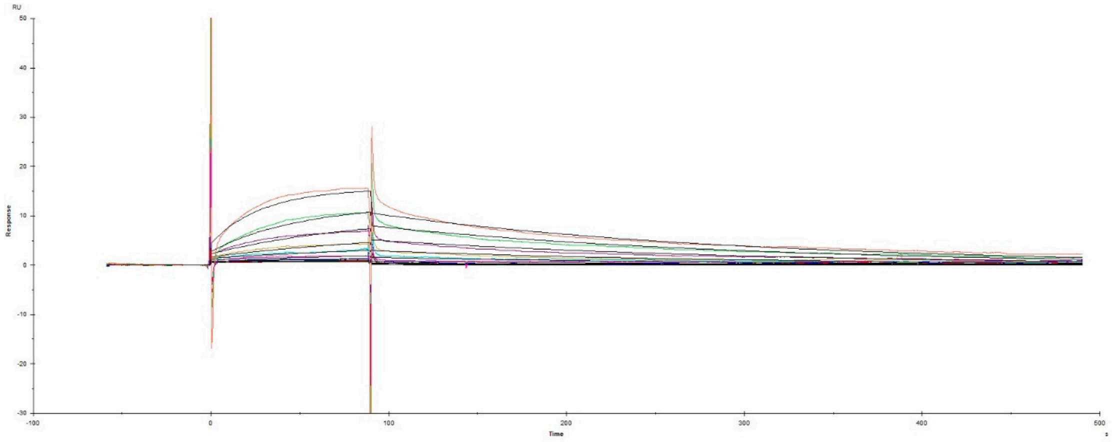

Figure S48. Sensorgram of compound 12 on BAG3-BD

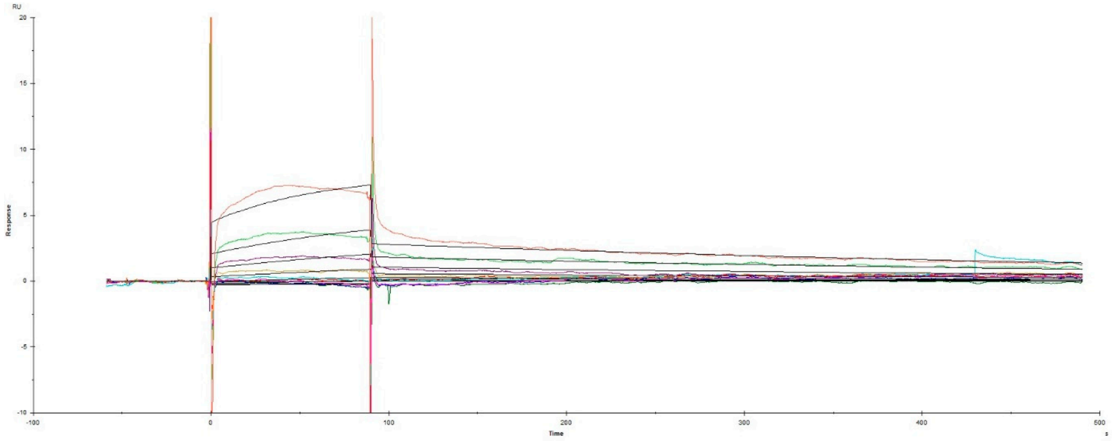

Figure S49. Sensorgram of compound 14 on BAG3-BD

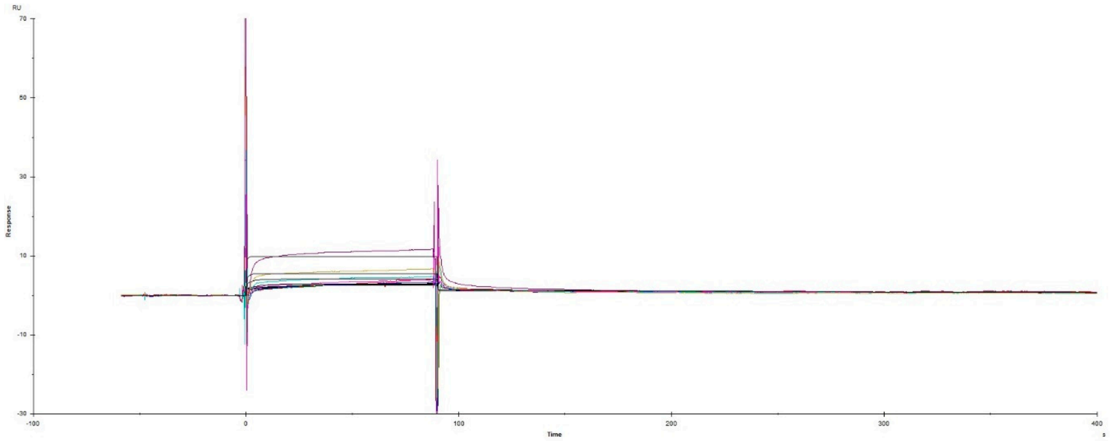

Figure S50. Sensorgram of LK4 on BAG3-BD

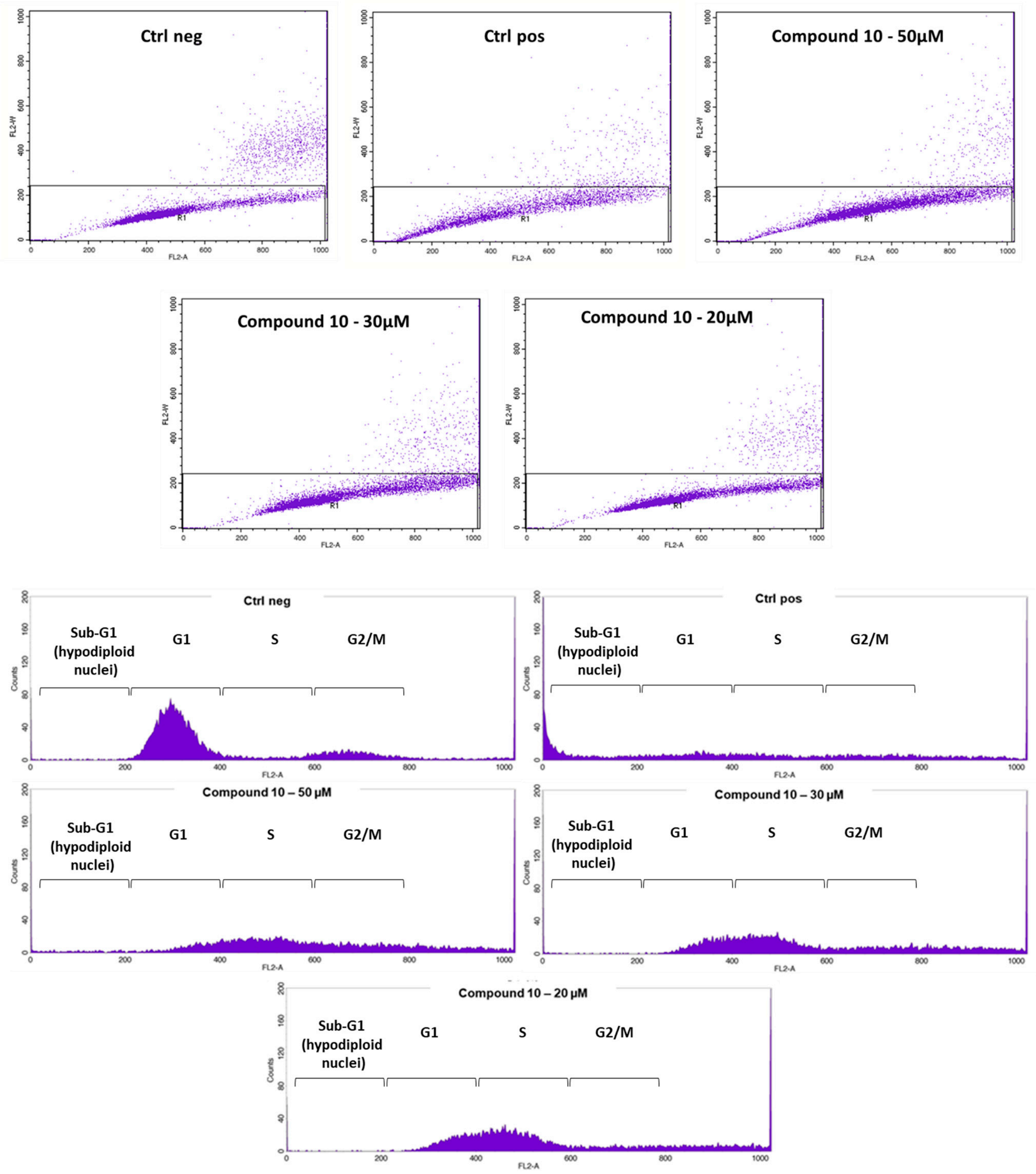

Figure S51. FACS plots and histograms for cell cycle analysis.

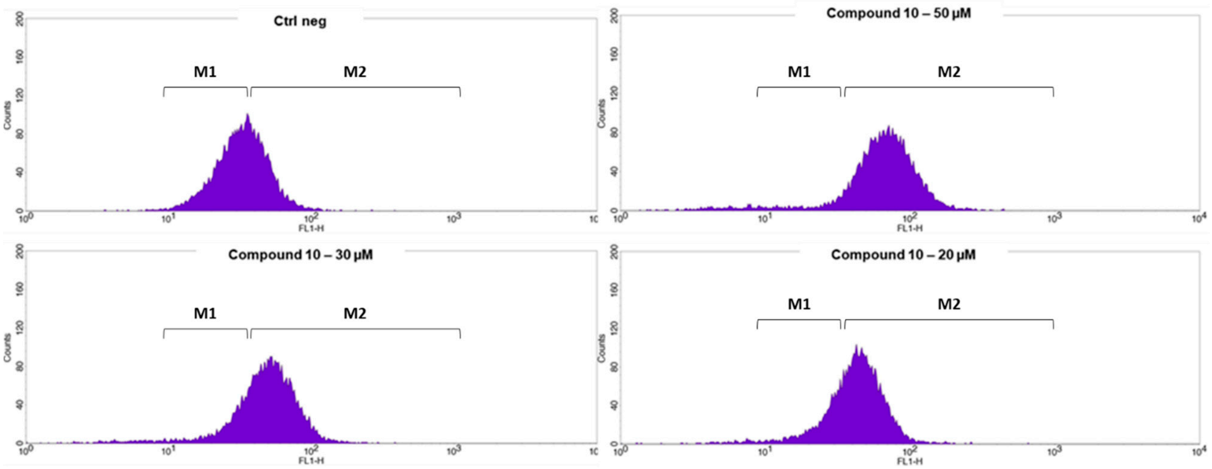

Figure S52. FACS histograms for caspase 3 analysis.

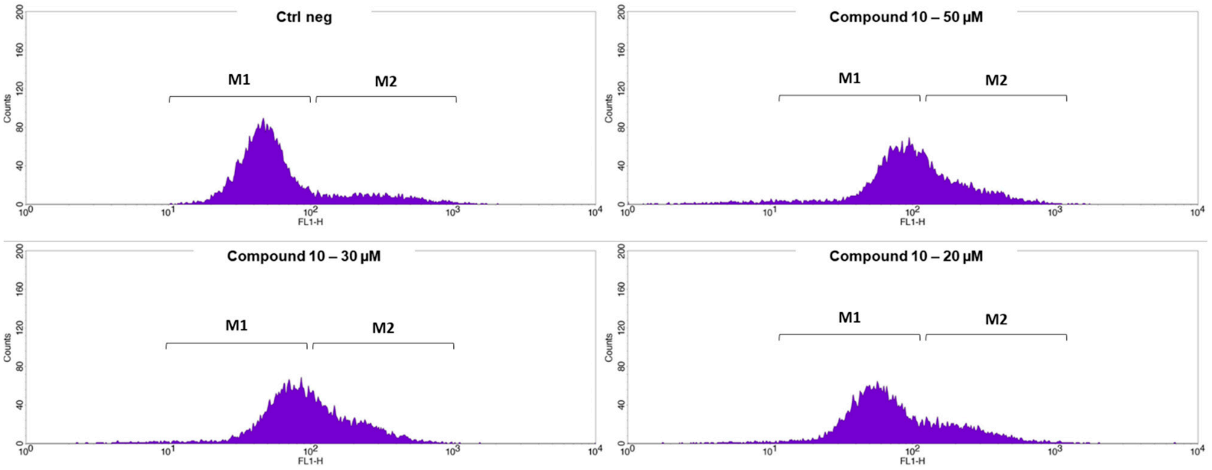

Figure S53. FACS histograms for caspase 9 analysis.

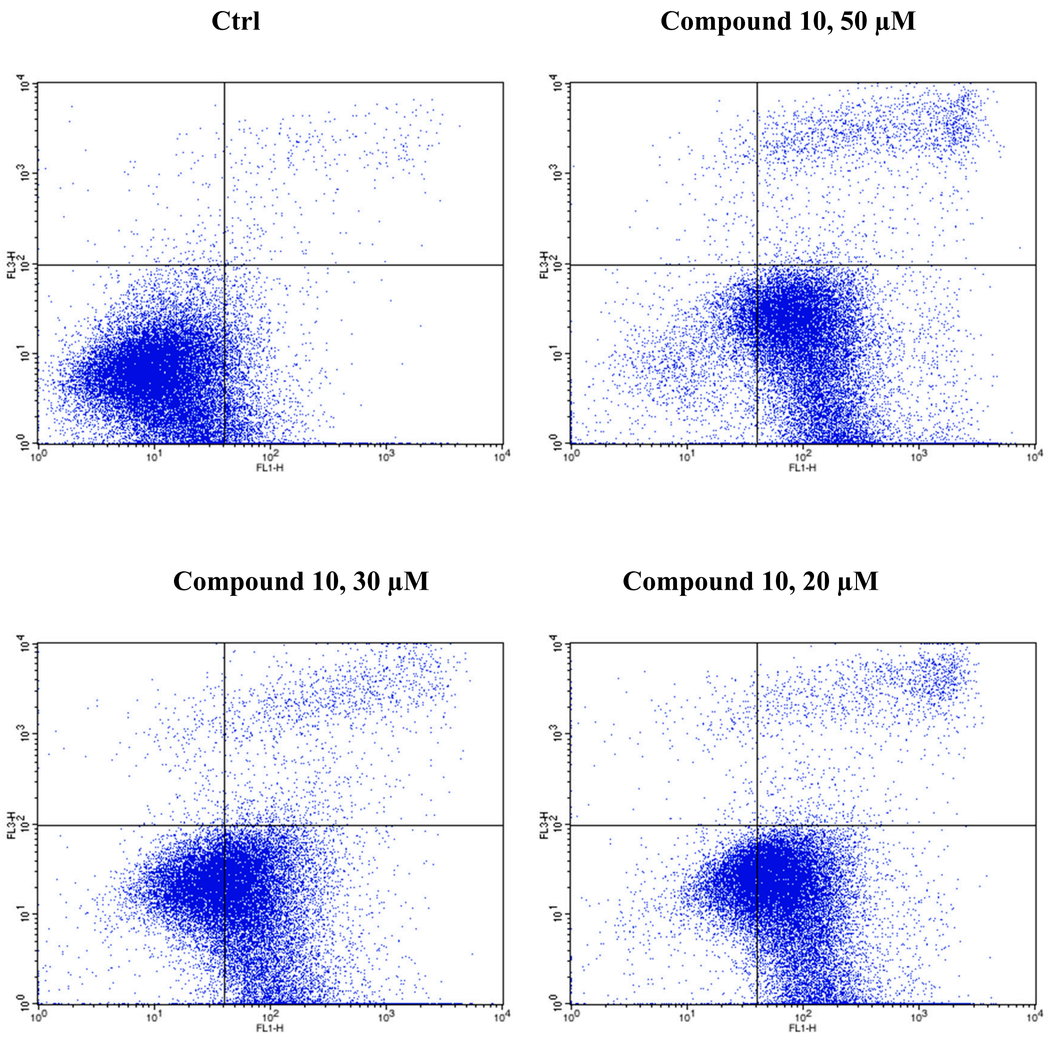

Figure S54. Data plot of apoptotic analysis of compound 10 on HeLa cells using AnnexinV-FITC/PI staining.

Cleaved caspase 3

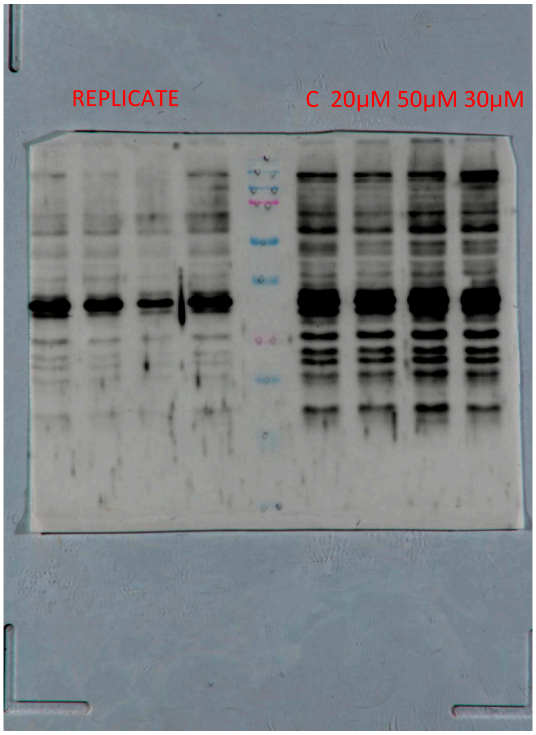

Caspase 3

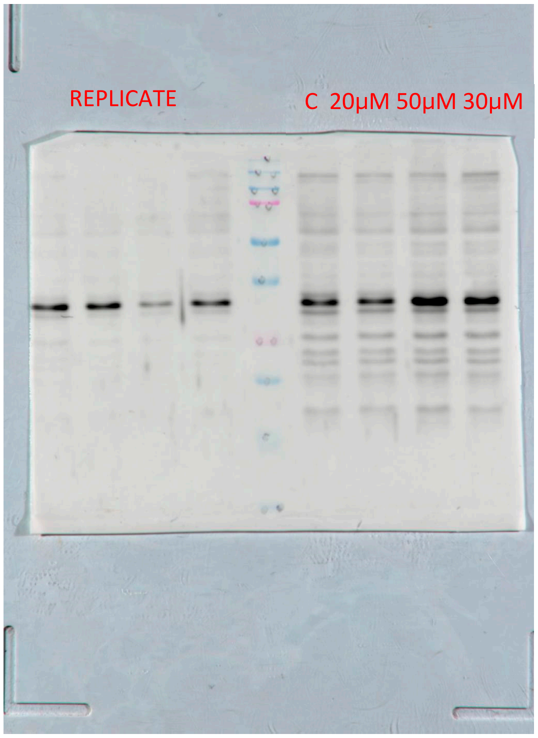

β-Tubulin

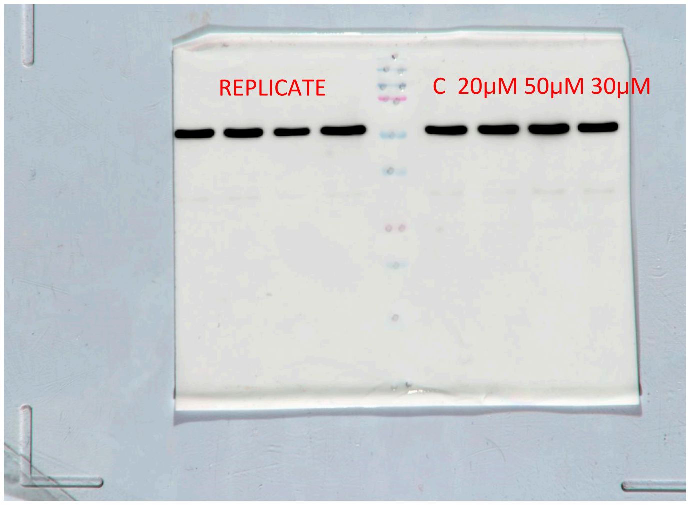

Figure S55. Western blots raw data of caspase 3 and β-tubulin after treatments with compound 10 on HeLa cells.
